# Multilevel Diabetes Prevention Interventions to Address Population Inequities in Diabetes Risk: Scoping Review

**DOI:** 10.2196/70267

**Published:** 2025-08-25

**Authors:** Reshma Patel, Kathy Kornas, David Gerstle, Lori M Diemert, Laura C Rosella

**Affiliations:** 1 Dalla Lana School of Public Health, University of Toronto, 155 College Street, Toronto, ON, M5T1P8, Canada, 1 4169786064; 2 University of Toronto Mississauga Library, University of Toronto, Mississauga, ON, Canada; 3 Institute for Better Health, Trillium Health Partners, Mississauga, ON, Canada; 4 Laboratory Medicine and Pathobiology, Temerty Faculty of Medicine, University of Toronto, Toronto, ON, Canada

**Keywords:** type 2 diabetes, multilevel intervention, prevention, health equity, review

## Abstract

**Background:**

Type 2 diabetes risk is disproportionately higher among structurally marginalized communities, partly due to influences from social, economic, and environmental determinants of health. Individual-level diabetes prevention strategies address proximal factors, such as modifiable behaviors, often overlooking the role of multilevel socioecological factors that contribute to diabetes risk and inequities. Multilevel diabetes prevention interventions involve actions that address multiple health determinants across the individual, community, and systemic levels of influence, offering a promising approach to reducing inequities in diabetes risk.

**Objective:**

This scoping review aimed to systematically map the types of health determinants addressed in multilevel diabetes prevention interventions that have been implemented for addressing population inequities in diabetes risk and to describe what evidence exists regarding their effectiveness.

**Methods:**

A comprehensive literature search was conducted in PubMed, CINAHL, MEDLINE, Embase, Web of Science, and gray literature sources (websites of government agencies and local/international nongovernmental health organizations) for studies published from the year 2000 to 2024. The research team developed a conceptual framework to guide the scoping review and define multilevel interventions for eligibility. Eligibility criteria included studies focusing on multilevel diabetes prevention interventions targeting diabetes relevant risk factors at more than one level of influence (micro, meso, and macro) and where intervention outcomes were reported. Data extraction included study characteristics, intervention target populations and coverage, targeted health determinants, and intervention outcomes and was completed by 2 independent reviewers. Data synthesis involved mapping health determinants addressed by each multilevel intervention according to our conceptual framework and a narrative synthesis of findings on themes corresponding to intervention types and reported outcomes.

**Results:**

Of 7813 articles retrieved, a total of 25 studies met the inclusion criteria. Interventions consisted of targeted interventions for high-risk populations (n=7), environmental-based interventions (n=7), and community-based interventions (n=11). Most interventions addressed health determinants at 2 levels (micro and macro) (14/25, 56%) or 3 levels (micro, meso, and macro) (11/25, 44%). All studies reported on proximal outcomes, most frequently on weight, physical activity, and dietary behaviors. One-third (8/25, 32%) of studies reported outcomes on changes in metabolic risk. None of the studies reported on equity outcomes related to changes in population inequities in diabetes incidence. Only 8% (n=2) of studies reported an equity outcome that captures disparities in a diabetes risk factor level between disadvantaged and advantaged population groups.

**Conclusions:**

Our review identified a research gap in that outcomes on population inequities in diabetes risk have not been consistently measured in multilevel diabetes prevention interventions, and the impact of these interventions on reducing population inequities in diabetes incidence is not consistently examined or reported. Future research should prioritize equity outcomes in evaluations of multilevel diabetes prevention interventions and emphasize impacts on disadvantaged populations and population inequities.

## Introduction

Preventing type 2 diabetes is a priority for health systems worldwide, as reflected by the United Nations Global Sustainable Development Goal, which emphasizes good health and well-being and underscores the importance of preventing chronic conditions [1]. Globally, there is a high burden of type 2 diabetes [2], in addition to persistent inequities in diabetes risk among population groups, disproportionately impacting racialized populations, low-income, and marginalized communities [3-5]. These inequities are more pronounced at younger ages; for example, South Asian and Black ethnic groups have been found to have higher type 2 diabetes prevalence among younger age groups (19‐39 years), compared with White European populations [6].

Multiple factors at the individual, health system, neighborhood, and socioeconomic levels (eg, obesity, unhealthy diets, low physical activity, health care access, income, neighborhood, and food environments) contribute to the inequities in diabetes risk [7]. For example, the association between obesity and diabetes varies in different ethnic groups. South Asians, for instance, have a higher burden of diabetes at lower BMI levels compared with White populations [3,8]. Built environments have been shown to differentially impact diabetes risk in different population groups. For instance, in Ontario (Canada), prediabetes incidence was found to be lower in highly walkable neighborhoods; however, the association varied across ethnic groups, with South Asians, South East Asians, Sub-Saharan Africans, and Caribbeans benefiting less from walkable areas than Western Europeans [9].

Historically, diabetes prevention efforts have concentrated on individual-level interventions that aim to address modifiable lifestyle and clinical risk factors [10]. These strategies—such as lifestyle modifications, pharmacotherapy, and metabolic surgery for those at high risk—have demonstrated effectiveness in reducing diabetes risk and play an important role in diabetes prevention [11]. However, interventions focused on proximal factors, such as behavior change, can widen inequities because they do not consider social and environmental contexts that support or impede lifestyle changes [12]. Furthermore, structural and social determinants that contribute to inequities in diabetes risk cannot be addressed at the individual level alone [13]. It is increasingly being recognized that there is no one-size-fits-all solution for diabetes prevention that is effective for all populations and achieves health equity [14].

Multilevel interventions that address social determinants and individual-level risk factors across multiple levels of influence (micro, meso, and macro) are increasingly recognized as one solution to reduce inequities in diabetes risk and improve population health [14-16]. Multilevel interventions are strategies that aim to improve health outcomes by targeting at least 2 levels of influence, from the individual, community, and structural/environmental/policy context [15]. The individual level refers to actions directed at individuals, the community level refers to actions that target institutions and the community context, and the structural/policy level refers to actions directed at the social, policy, and environmental context [17].

Previous reviews of multilevel diabetes prevention interventions that have been implemented in different community and social settings have focused on workplace wellness programs [18], faith-based interventions to address obesity [19], population-wide programs that target the built and food environments [20], and multilevel interventions targeted for Native peoples [21]. This scoping review was conducted to systematically map the range of multilevel diabetes prevention interventions on addressing inequities in diabetes risk. Our review is unique because of its broader population-based focus and objective of gaining insight into what health determinants and equity factors across the micro, meso, and macro levels are addressed in multilevel diabetes prevention interventions. In addition, we focused on describing evidence on the impact of these interventions on reducing inequities in diabetes risk.

Specifically, we aimed to answer the research question: What multilevel diabetes prevention interventions have been implemented to address population inequities in diabetes risk, and what evidence exists regarding their effectiveness? Our review pursued three objectives: (1) to map the types of health determinants and equity factors addressed across the levels of influence (micro, meso, and macro) in multilevel interventions for diabetes prevention; (2) to identify the target population groups in multilevel interventions for diabetes prevention in the identified studies, including intervention coverage; and (3) to describe the types of proximal and distal outcomes reported in the identified studies for the changes in risk factors for diabetes, population risk distribution (changes in diabetes risk), and changes in population inequities in diabetes risk.

## Methods

### Scoping Review and Theoretical Framework

The review methodology followed the framework of Arksey and O’Malley [22] and the Joanna Briggs Institute [23], and adhered to the PRISMA-ScR (Preferred Reporting Items for Systematic reviews and Meta-Analyses extension for Scoping Reviews) guidelines (Checklist 1). Scoping reviews are specifically useful for reviewing health research evidence and identifying research gaps [24] and therefore align with the goals of our review. The review protocol was registered on Open Science Framework [25].

We developed a conceptual model based on 2 theoretical frameworks to guide the scoping review. The social ecological model [26] is a commonly used framework for describing factors of influence at the micro, meso, and macro levels and has been previously applied to understand the multiple levels of influence for diabetes prevention [27], demonstrating its applicability to multilevel diabetes prevention interventions. The micro level consists of intrapersonal (individual-level) factors (eg, health behaviors) and interpersonal factors (eg, small group interactions, social support). The meso level consists of organizational structures and processes (eg, health care institutions, workplace health programs, and activities in the school setting), and community factors (eg, characteristics and resources in communities). The macro level represents socioeconomic conditions, environmental factors, and policy influences (eg, built environment, housing, and food supply). The wider determinants of health model [28] was chosen as it is a widely used social determinants framework to understand the elements essential to achieve health equity and identify specific factors that influence health across different levels, including socioeconomic and structural factors, community and social factors, and intrapersonal lifestyle factors. A previous review identified social determinants of health associated with diabetes risk [29], which we integrated into our conceptual model. A comprehensive table outlining the conceptual model is available in Multimedia Appendix 1.

### Search Strategy

We developed the search strategy with a research librarian (DG) from the University of Toronto. We also consulted with an additional librarian through the Peer Review for Electronic Search Strategies (PRESS). PRESS evaluation is widely advocated by librarians, who contribute to knowledge syntheses to draw on additional expertise in developing keywords, identifying subject headings (eg, medical subject heading terms), and refining lines of the strategy [30]. Our PRESS submission was facilitated by the research librarian through the University of Toronto Libraries’ Knowledge Syntheses Community of Practice.

We searched indexed databases of peer-reviewed literature, gray literature indexed in web sources, and manual searches of reference lists from related published reviews. To concentrate on contemporary research on multilevel interventions for diabetes prevention, we restricted our search to publications from the year 2000 onward. We considered this an appropriate cut-off given that key frameworks and influential guidance for developing and evaluating complex interventions have primarily been published after the year 2000 [31,32]. We limited our search to high-income economies, using filters based on the World Bank Country and Lending Groups [33]. We excluded publications in non-English languages without available English translations, as well as letters, editorials, opinion pieces, book chapters, and conference abstracts.

The peer-reviewed literature search was conducted in May 2024 across 5 electronic databases: MEDLINE (OVID platform), Embase (OVID platform), CINAHL, Scopus, and Web of Science. Gray literature web sources from government agencies and local/international nongovernmental health organizations were searched in July 2024 by applying search strings developed by the research librarian. If search strings were not supported, then we manually reviewed the first 10 pages of relevant websites. Specifically, we searched the websites of the World Health Organization, the Centers for Disease Control and Prevention, the Organization for Cooperation and Development, the Public Health Agency of Canada, Health Canada, Public Health Ontario, and Peel Public Health. Search terms corresponded to the concepts of type 2 diabetes, multilevel, and prevention. The finalized search strategy implemented in MEDLINE and the search strings used on websites are provided in Multimedia Appendix 2. Identical searches were customized for the remaining peer-reviewed databases. Finally, the reference list of related reviews, identified from the search of peer-reviewed databases, was manually searched for additional articles for inclusion [34-38].

### Eligibility Criteria

In addition to the criteria described above for timeframe, geography, language, and publication type, the following population, intervention, and outcome eligibility criteria were applied in screening. Studies were included where the study population/target group for the intervention included individuals without diabetes (youth and adults). Studies were excluded where the study population/target group for the intervention included individuals with diabetes (ie, type 1, type 2, and gestational). Studies were included if intervention outcomes were reported. Studies that focused on describing the development or implementation of a multilevel intervention were excluded.

Studies were excluded if the intervention was unrelated to diabetes prevention. For this review, we considered an intervention applicable to diabetes prevention if its actions aimed to reduce diabetes risk or diabetes-relevant risk factors (eg, physical activity, BMI, and diet). Studies were included where the intervention aimed to target diabetes-relevant risk factors at more than one level of influence (ie, micro, meso, and macro). Studies on interventions that targeted one level of influence (eg, an education session in a group setting, enhancing social support [interpersonal] to improve diet [intrapersonal]) were considered one level of influence (the micro level in this example) and excluded from our review. Similarly, interventions were excluded where activities took place at multiple levels, but the target for change took place at only one level. For example, a mass media campaign seeking to promote physical activity would be excluded if the target for change took place only at the micro level.

In the process of screening studies, we identified existing reviews that synthesized findings on specific types of multilevel interventions related to diabetes prevention. Thus, we modified our original protocol by expanding our exclusion criteria for the following types of interventions that were the subject of an existing review: multilevel diabetes prevention interventions for Native and Indigenous Peoples [21]; diabetes prevention interventions implemented at the workplace setting [18,39,40]; built environment interventions related to investment in park/recreational areas, point-of-decision prompts to encourage stair use and active travel interventions [20]; food environment interventions related to economic measures (sugar-sweetened beverage prices, fast food prices, fruit and vegetable prices) and policy measures for food labeling or changing the layout/food provided at grocery stores [20]; interventions for physical activity in after school hours childcare settings (eg, timetabling changes, provision of equipment for active play, and changes in policies at regional or national level) [37]; school-based interventions aimed at reducing sedentary behavior (eg, changes to classroom design, such as sit-to-stand desks, and changes to curriculum) [41]; and church-based interventions targeted for African American and Latino communities [19]. A summary of the findings from each review on excluded multilevel interventions can be found in Multimedia Appendix 3 [18-21,37,39-41]. A summary of the scoping review inclusion and exclusion criteria can be found in Multimedia Appendix 4.

### Screening Process

Abstract and full-text screening was done through Covidence (Veritas Health Innovation Ltd), a software for managing and streamlining reviews. First, 2 reviewers (RP and KK) independently screened all identified papers for relevance by title and abstract and categorized them as “include,” “exclude,” or “inconclusive.” Categorization disagreements were resolved through discussion against the eligibility criteria. Next, the full text of all articles marked as “include” and “inconclusive” was independently reviewed by 2 reviewers, and disagreements were resolved through discussion for a decision regarding the final inclusion.

### Data Extraction

All data were extracted into a standardized data extraction template created using the extraction feature on Covidence. Specifically, 2 reviewers (RP and KK) acted as primary reviewers to abstract the data for half of the articles and as secondary reviewers to verify the data for the other half of the articles. Disagreements were addressed through discussion between reviewers. Data extraction items were informed by our conceptual framework. From the included studies, we extracted study characteristics (year of publication, publication type, and country of publication), intervention characteristics (setting, description, target population, coverage, levels of influence targeted, diabetes relevant health determinants targeted, and equity factors targeted), and intervention outcomes (changes in diet, physical activity, BMI/weight, blood pressure, metabolic risk, and inequities in diabetes risk). Inequity was defined as a health difference that adversely affects disadvantaged populations on the basis of a higher incidence of disease.

### Synthesis

Quantitative data were synthesized descriptively to summarize study characteristics, types of multilevel interventions, target populations, and types of outcomes. We grouped studies based on intervention type. Health determinants targeted by each multilevel intervention were mapped according to our conceptual framework. A narrative synthesis was used to analyze qualitative themes corresponding to intervention types and intervention outcomes.

## Results

### Overview

**Figure 1.**
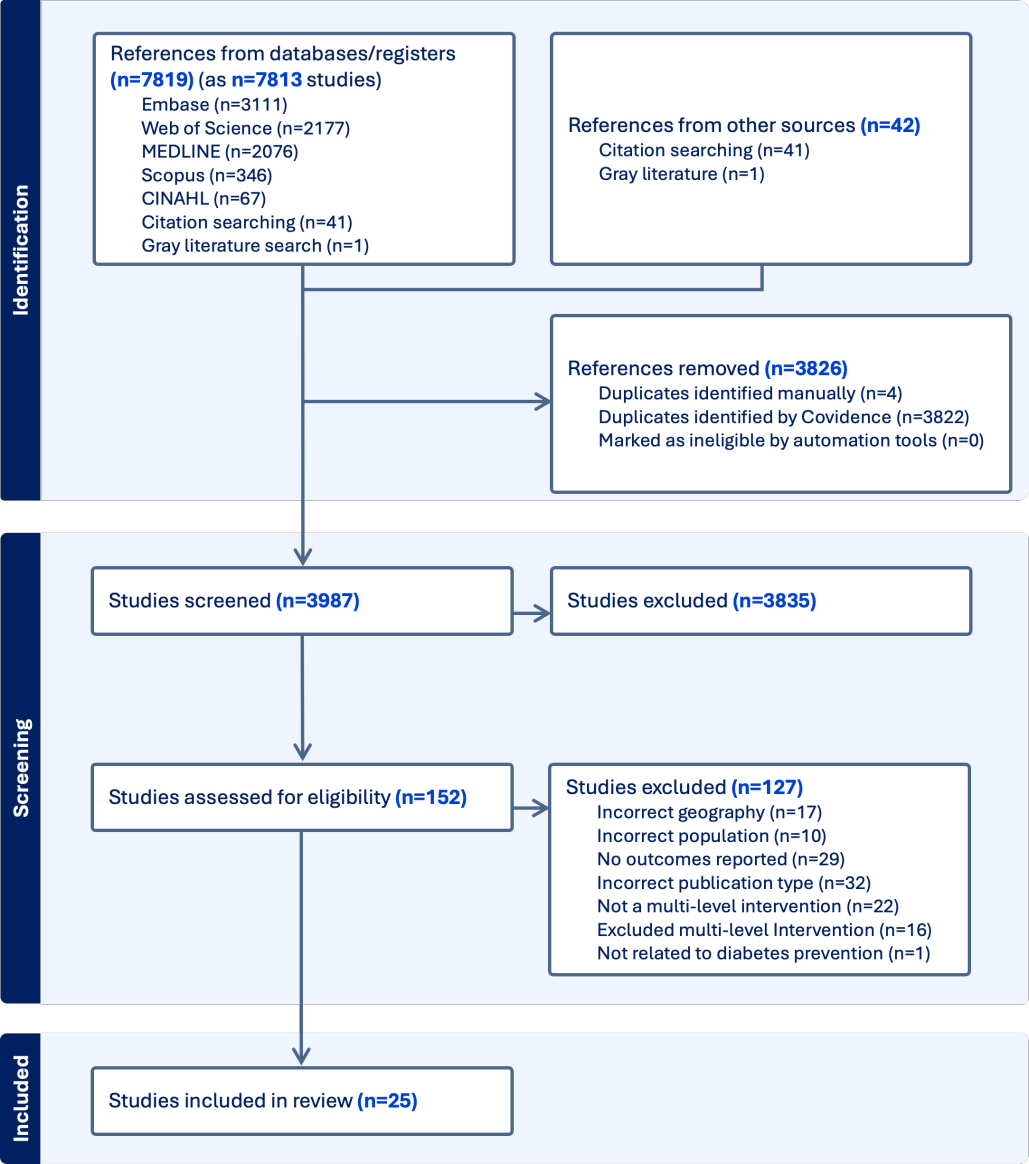
Preferred reporting items for systematic reviews and meta-analyses flowchart of the article screening process.

We initially retrieved 7813 articles for screening, which were imported into Covidence. After removing duplicates, 3987 articles remained for further evaluation. Following the abstract screening, 3835 articles were excluded, and an additional 127 articles were excluded after full-text screening. Our review includes 25 studies related to multilevel interventions for the prevention of type 2 diabetes. The detailed screening process is illustrated in Figure 1.

**Figure 2.**
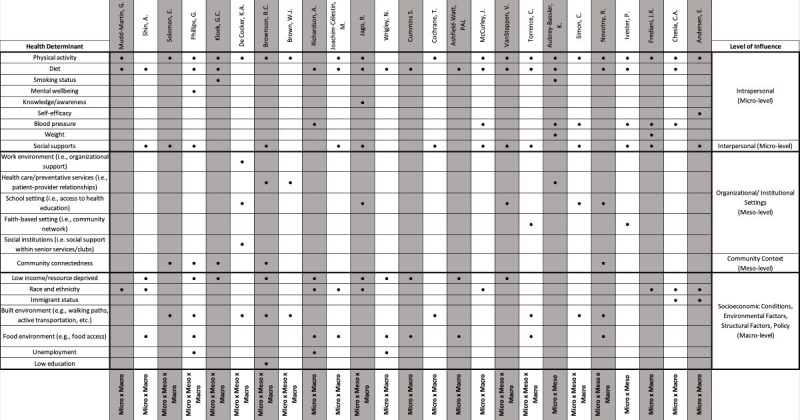
Health determinants targeted by multilevel interventions for diabetes prevention [42-66].

The included studies described interventions targeting diabetes-free or undiagnosed individuals, spanning 2 or 3 levels of influence: micro, meso, and macro. Given the inclusion criteria and multilevel nature of this review, it should be noted that all interventions addressed at least 2 health determinants across at least 2 levels of influence (micro, meso, and macro). Specifically, most interventions addressed health determinants at the micro and macro levels (12/25, 48%) or all 3 levels (micro, meso, and macro) (11/25, 44%), with the remaining interventions focusing on health determinants at the micro and meso levels (2/25, 8%) (Figure 2).

### Characteristics of Included Studies

Most studies were conducted in the United States (12/25, 48%), and the remainder were from Canada (1/25, 4%), the United Kingdom (6/25, 24%), Australia (1/25, 4%), and several European countries including the Netherlands, Belgium, Finland, Greece, Spain, Hungary, France, and Norway (5/25, 20%). The publication dates were evenly distributed, with all studies published between 2003 and 2022. The studies used various methodological designs, such as randomized controlled trials (9/25, 36%), quasi-experimental studies (9/25, 36%), pretest/posttest designs (3/25, 12%), a cohort study (1/25, 4%), an evaluation (1/25, 4%), one-arm pilot clinical trial (1/25, 4%), and a community case study (1/25, 4%). The study characteristics are presented in Table 1.

**Table 1.** Study characteristics of diabetes prevention articles included in the scoping review (2000‐2024) (n=25).

Characteristic	Number of articles, n (%)
Country	
United States	12 (48)
United Kingdom	6 (24)
Australia	1 (4)
Belgium	1 (4)
Canada	1 (4)
France	1 (4)
Netherlands	1 (4)
Norway	1 (4)
Multicountry (within Europe)	1 (4)
Year published	
2003‐2007	7 (28)
2008‐2012	5 (20)
2013‐2017	7 (28)
2018‐2022	6 (24)
Study design	
RCT^a^	9 (36)
Quasi-experimental	9 (36)
Pretest/posttest	3 (12)
Cohort study	1 (4)
Evaluation	1 (4)
One-arm pilot trial	1 (4)
Community case study	1 (4)

a RCT: randomized controlled trial.

### Types of Multilevel Interventions for Diabetes Prevention

We grouped the intervention types identified in our review into 3 main categories: targeted interventions for high-risk populations (n=7), environmental-based interventions (n=7), and community-based interventions (n=11). A detailed summary of each intervention is provided in Multimedia Appendix 5 [42-66].

Targeted interventions for high-risk populations included a range of educational and culturally adapted programs. Interventions that addressed health determinants at the micro-macro levels featured peer-led initiatives tailored for Latinx communities [42-44], a recreational soccer program tailored for Hispanic men [45], a lifestyle education program tailored for Chinese Americans [46], and a lifestyle intervention tailored for Pakistani immigrant men [47]. One multicomponent intervention for US-affiliated Pacific Islanders addressed health determinants at the micro-meso-macro levels [48].

Environmental-based interventions targeted changes in the built or food environments within communities. These interventions addressed health determinants across micro-macro levels. For example, efforts to increase food access and availability were implemented through nutrition programs in recreation centers, corner stores, and carry-out restaurants in Baltimore, United States [49], as well as through initiatives aimed at improving access to food retail in food deserts [50-53]. Additionally, built environment interventions that addressed health determinants at the micro-meso-macro levels included enhancements to footpaths with signage promoting walking [54] and changes implemented within institutions, such as the integration of physical activity programs in schools to reduce barriers in adopting an active lifestyle for children [55].

Community-based interventions spanned various communities defined by region or group membership in an institutional setting and addressed health determinants at multiple levels. One intervention targeted the micro-macro levels by increasing physical activity programming in a materially deprived community in the United Kingdom [56]. Others targeted the micro-meso levels, such as a wellness program for overweight and obese adults within church congregations [57] and a patient-practitioner program for individuals in remote and rural areas [58]. Finally, community-based interventions that targeted health determinants across micro-meso-macro levels included diverse programs promoting physical activity and community connectedness in low-income or resource-deprived communities in Devon County (United Kingdom) [59], the United Kingdom [60], the Netherlands [61], Ghent (Belgium) [62], and the United States [63]. Other interventions that addressed health determinants across the micro-meso-macro levels included physical activity and nutrition education programs delivered in the school setting, focusing on school-aged children and school teachers [64,65], and a family-based cooking and eating smart program with participants of faith-based organizations [66].

### Target Populations in Multilevel Interventions for Diabetes Prevention

Across the included studies, 21 (84%) multilevel interventions targeted disadvantaged communities or population groups characterized by low income, resource deprivation, rural and remote locations, and racialized populations. A total of 4 (16%) multilevel interventions targeted the whole population (eg, an entire community). Targeted interventions for high-risk populations were aimed at racialized groups (100%). Environmental-based interventions primarily targeted low-income or resource-deprived communities (4/7, 57%) and a racialized group (1/7, 14%). Last, community-based interventions targeted low-income or resource-deprived communities (6/11, 55%) and rural/remote communities (3/11, 27%). These details are highlighted in Table 2.

**Table 2.** Population targets in multilevel diabetes prevention interventions included in the scoping review (2000‐2024) according to intervention type.

Intervention type and target population	Number of articles, n (%)
Targeted interventions for high-risk populations (n=7)	
Racialized group (Latinx, Chinese immigrants, Pakistani immigrants, and Pacific Islanders)	7 (100)
Environmental-based interventions (n=7)	
Low-income/resource-deprived	4 (57)
Racialized group (African American youth)	1 (14)
Whole population	2 (29)
Community-based interventions (n=11)	
Low-income/resource-deprived	6 (55)
Rural/remote community	3 (27)
Whole population	2 (18)

### Health Determinants Targeted in Multilevel Interventions for Diabetes Prevention

Figure 2 illustrates the health determinants targeted in each multilevel intervention. Among the 25 multilevel interventions, all (n=25, 100%) targeted health determinants at the microlevel. Physical activity (20/25, 80%) and diet (17/25, 68%) were the most common intrapersonal health determinants, while social support from family, friends, or peers (14/25, 56%) was the most common interpersonal health determinant.

A total of 13 of the 25 interventions (52%) addressed health determinants at the mesolevel. Using the school setting for health education and access to health services to promote a healthy school environment (4/25, 16%), as well as leveraging patient-provider relationships within primary care, were the most frequently addressed health determinants at the organization and institutional level. Additionally, leveraging community social supports (5/25, 20%) was the most common health determinant addressed at the community level.

Finally, 23 of the 25 interventions (92%) addressed macrolevel health determinants. These interventions often targeted low-income or economically deprived neighborhoods and communities (10/25, 40%), racialized groups with disproportionate diabetes risk (8/25, 32%), or aimed to enhance the community’s built environment (9/25, 36%) and food environment (8/25, 32%).

### Intervention Coverage

Intervention coverage was reported in 76% (n=19) of the studies. For interventions targeting high-risk individuals and community-based interventions, coverage was measured based on average session attendance, with program participants as the denominator. Coverage for targeted interventions ranged from 60% to 100%, while for community-based interventions, it ranged from 5.2% to 83.5%. In environmental-based interventions, coverage was primarily measured by activity retention, with the denominator being participants who completed the activity. Coverage for these types of interventions ranged from 18% to 70%. Notably, 6 (24%) studies did not report on coverage. Details on intervention coverage for each study are provided in Multimedia Appendix 5 [42-66].

### Outcomes Reported in Multilevel Interventions for Diabetes Prevention

This review focused on outcomes reported in studies across 3 main domains: changes in diabetes risk factors (weight/BMI, physical activity, diet, and blood pressure), changes in metabolic risk, and changes in inequities in diabetes risk (Table 3). While all studies included in the review reported on proximal outcomes related to changes in diabetes risk factors (n=25, 100%), fewer addressed distal outcomes, with only 32% (n=8) assessing changes in metabolic risk. None of the studies reported on changes in population inequities in diabetes incidence, and only 8% (n=2) reported an equity outcome related to a change in the difference in a diabetes risk factor level between disadvantaged and advantaged population groups. A detailed summary of the outcomes reported in each multilevel intervention is presented in Multimedia Appendix 6 [42-66].

**Table 3.** Measured outcomes reported in studies of multilevel diabetes prevention interventions included in the scoping review (2000‐2024) by intervention type.

Intervention type, measured outcomes, and group reported on	Number of articles, n (%)
Targeted intervention for high-risk population (n=7)	
Weight/BMI	
Total population	N/A^a^
Population group	5 (71)
Both	N/A
Physical activity levels	
Total population	N/A
Population group	6 (86)
Both	N/A
Diet	
Total population	N/A
Population group	5 (71)
Both	N/A
Blood pressure	
Total population	N/A
Population group	3 (43)
Both	N/A
Metabolic risk	
Total population	N/A
Population group	4 (57)
Both	N/A
Inequity	
Total population	N/A
Population group	1 (14)
Both	N/A
Environmental-based intervention (n=7)	
Weight/BMI	
Total population	1 (14)
Population group	2 (29)
Both	0 (0)
Physical activity levels	
Total population	0 (0)
Population group	1 (14)
Both	1 (14)
Diet	
Total population	3 (43)
Population group	0 (0)
Both	1 (14)
Blood pressure	
Total population	1 (14)
Population group	1 (14)
Both	0 (0)
Metabolic risk	
Total population	1 (14)
Population group	1 (14)
Both	0 (0)
Inequity	
Total population	0 (0)
Population group	1 (14)
Both	0 (0)
Community-based intervention (n=11)	
Weight/BMI	
Total population	1 (9)
Population group	—^b^
Both	0 (0)
Physical activity levels	
Total population	6 (55)
Population group	—
Both	4 (36)
Diet	
Total population	3 (27)
Population group	—
Both	2 (18)
Blood pressure	
Total population	2 (18)
Population group	—
Both	0 (0)
Metabolic risk	
Total population	1 (9)
Population group	—
Both	1 (9)
Inequity	
Total population	0 (0)
Population group	—
Both	0 (0)

a Not applicable; targeted interventions for high-risk populations were not population-wide interventions and, therefore, did not report outcomes for the total population.

b Not reported.

Due to the variation in the target populations of the interventions, some studies reported outcomes for the entire population (eg, whole community) (10/25, 40%), while others reported outcomes on specific population subgroups (eg, sex, age, and ethnicity) (9/25, 36%). A smaller number of studies reported outcomes for both the total population and specific subgroups (6/25, 24%).

Across studies, outcomes were measured in different ways (Multimedia Appendix 6 [42-66]). For example, physical activity was measured by walking scores [62,63], self-reported physical activity scores [42,44,66], or moderate to vigorous physical activity sessions per week [48,64,65].

Targeted interventions for high-risk populations reported outcomes for population groups, particularly on changes in risk factors such as weight/BMI (5/7, 71%), diet (5/7, 71%), physical activity levels (6/7, 86%), and changes in metabolic risk (4/7, 57%). Studies reported significant changes in physical activity [42,43,46,47] and weight [43,45,46,48], while changes in metabolic risk [44-46] and diet [42,45,48] were often insignificant. For instance, one educational program that aimed to reduce cardiovascular risk among Latinas demonstrated increased physical activity levels postintervention, but no significant changes in diet, as measured by the Health-Promoting Lifestyle Profile subscale scores [42]. Another culturally adapted healthy eating and educational nutrition program for Latinas found a reduction in weight, increased physical activity, and higher consumption of fiber-rich foods postintervention [43]. A peer-led, culturally tailored community-based program targeting Latina women reported improved healthy eating scores; however, HbA_1C_ levels remained unchanged [44]. Among Hispanic males, a recreational soccer program led to a reduction in weight and diastolic blood pressure, but there were no notable changes in diet measured by total energy intake, fat consumption, or fruit and vegetable intake [45]. Similarly, a culturally adapted lifestyle education program for Chinese immigrant adults in the United States reported reduced BMI, increased physical activity, improved dietary intake scores, and lower diastolic blood pressure, but fasting plasma glucose and HbA_1C_ levels remained unchanged [46]. Finally, a multicomponent physical activity and counseling program targeted to Pakistani immigrant men in Norway showed an increase in overall physical activity postintervention [47].

Environmental-based interventions generally reported outcomes for the total population and most frequently reported changes in risk factors such as weight/BMI (3/7, 43%) and diet (4/7, 57%). For example, a built-environment intervention that removed barriers to physical activity in schools and targeted African American youth found that normal weight students in the intervention group had a smaller increase in age- and gender-adjusted BMI over time compared with the control group. Similarly, overweight students in the intervention group also experienced a smaller increase in BMI than their control counterparts [55]. Additionally, a nutrition intervention aimed at improving the food environment in community centers and corner stores demonstrated a decrease in BMI-age percentiles in the youth intervention group compared with the control group [49]. However, in another food-environment intervention, the Healthy Food Financing Initiative, where supermarkets were implemented in food deserts, changes in BMI, fruit and vegetable intake, and HbA_1C_ levels in the population showed no significant improvements [50].

Community-based interventions frequently reported outcomes for the total population and primarily reported changes in risk factors, such as physical activity (19/21, 91%) and diet (5/11, 45%). For instance, one intervention that introduced nutrition programs in primary schools and provided information on healthy nutrition and lifestyles to adults resulted in higher fruit self-efficacy scores and increased fruit consumption in intervention neighborhoods but no changes in physical activity scores among participants [61]. Another intervention, which developed community-wide physical activity programs with input from the community, led to increased self-reported walking scores, though there were no significant changes in participants meeting walking recommendations [63]. In a third study, which introduced 38 different types of activities in accessible community spaces, participants reported being more active compared with 1 year earlier [56]. Last, a family-centered, multilevel ecological intervention designed to improve nutrition and physical activity habits resulted in an increased mean change in physical activity frequency and intensity, as measured by the Rapid Assessment of Physical Activity Score. However, no changes were observed in fruit and vegetable consumption scores [66].

### Equity Outcomes Reported in Multilevel Interventions for Diabetes Prevention

None of the 25 interventions reviewed reported outcomes related to changes in population inequities in diabetes incidence. Only 2 studies (8%) reported an equity-related outcome between disadvantaged and advantaged population groups, specifically a change in the absolute differences in diabetes risk factor levels by food security and socioeconomic status (SES) [43,55].

The first study, a culturally adapted, community health worker-led healthy eating and nutrition intervention, found that participants with food insecurity (FI) benefited as much as those without FI in terms of improved diet and physical activity [43]. On average, both groups significantly increased their consumption of fiber-rich foods at program completion compared with baseline [43]. Although fiber-rich food consumption decreased between the 8-week and the 3-month follow-up, it remained significantly higher than baseline levels (10.16, no FI; 11.15, yes FI; diff: −0.99). Importantly, the gap in fiber-rich food consumption between food-secure and food-insecure groups narrowed over time, suggesting a positive shift in equity for this dietary measure (12.82, no FI; 12.87, yes FI; diff:−0.05). Physical activity levels also improved significantly in both groups at the 8-week and the 3-month follow-up, but the food-insecure group benefited more. While there was a small difference in physical activity levels favoring the food-secure group at baseline (19.11, no FI; 18.69, yes FI; diff: 0.42), by the 3 mo follow-up, the food-insecure group had achieved even greater improvements, closing and reversing the initial gap in physical activity (25.91, no FI; 34.03, yes FI; diff: −8.12).

The second intervention, which involved implementing environmental changes within a school setting to promote active lifestyles among adolescents, demonstrated effectiveness in increasing physical activity and reducing BMI across both low and high SES groups. The results showed no significant interaction between SES and the intervention (*P*=.82) [55], indicating that the benefits were equally distributed among students regardless of socioeconomic background.

## Discussion

### Principal Results

This review systematically mapped multilevel diabetes prevention interventions, providing insights into targeted, environmental-based, and community-based intervention types. Specifically, the review describes the health determinants targeted across micro, meso, and macro levels of influence, and the key outcomes reported. A central finding is the limited evidence available on the impact of these interventions in reducing population inequities in diabetes risk, highlighting the need for more research examining equity outcomes of multilevel diabetes prevention interventions. Our results revealed a range of health determinants across micro, meso, and macro levels that are acted upon in multilevel interventions for diabetes prevention. We also observed that no single intervention addressed all health determinants that contribute to diabetes risk and inequities, emphasizing that there is no one-size-fits-all solution.

The review points to the necessity of identifying an effective combination of strategies and a tailored approach that meets the unique needs of communities and at-risk populations, aligning with the concept of precision diabetes prevention [67,68]. As part of a precision diabetes prevention approach, data collection and tools are critical to inform new policies, including which multilevel interventions alongside other prevention strategies would be effective in communities in reducing diabetes incidence and inequities. The need for data collection, integration, and analysis to improve diabetes prevention and inform policies has been echoed in recommendations by The Lancet Commission on Diabetes [69]. To ensure direct and more effective consideration of multilevel factors that contribute to diabetes risk in communities, intersectoral collaboration and community engagement are required, including public health, health systems, educational institutions, government bodies, community organizations, and community members. Ultimately, equitable diabetes prevention strategies require an understanding of the lived experience and sociocultural context of communities in order to identify the underlying causes of diabetes disparities and inform effective local strategies and tailoring of interventions to address inequities [14,70].

All studies in this review reported on proximal outcomes of multilevel diabetes prevention interventions, such as changes in weight, physical activity, diet, and blood pressure. Few studies reported on long-term outcomes, such as sustained metabolic health improvements or reductions in inequities in diabetes risk, reflecting a research gap. These findings align with past reviews of other diabetes prevention interventions that have highlighted evidence on short-term outcomes of behavior change (eg, improved diet and increased physical activity), but less frequently reported evidence on long-term population-based outcomes on diabetes incidence and equity [20,21,39,41,71]. This research gap reflects challenges in measurement, particularly because of the long timeframe that is expected from exposure to a preventive intervention and impact on long-term outcomes [72]. Given the time lag to detect long-term outcomes, evaluations of interventions often use short-term outcomes as indicators of long-term impact [73]. It can also be challenging to assess population-level outcomes in targeted multilevel interventions if data are only available on a subset of the population (ie, high-risk individuals). Future research should consider methodologies and tools that enable measurement of the longer-term impacts of multilevel diabetes prevention interventions, potentially through data linkages with population data sources, modeling studies, and mixed methods approaches that allow for qualitative insights into the context of how interventions shape equity outcomes over time. Finally, given that interventions are typically assessed based on the goals they are designed to achieve [74], the gap in evidence on equity highlights the importance of identifying the intended equity impacts of multilevel interventions.

Determining the population health and equity impacts of multilevel diabetes prevention interventions requires considering the relative benefit of the intervention and the expected intervention coverage [75]. Our review demonstrated that population targets and intervention coverages in the included studies varied widely, and there was inconsistent reporting on intervention coverage across studies. Certain communities may experience barriers that result in lower uptake or differential benefits from health-promoting interventions or policies, which can result in the unintended effect of widened health disparities. Engaging the community, health care providers, and other stakeholders during the design and implementation phases may enhance the relevance and success of multilevel interventions [76]. This further endorses the precision diabetes prevention approach, which stresses the importance of tailoring interventions to local community needs and ensuring equitable uptake.

### Study Limitations

The scoping review does not present a comparative effectiveness of the different types of multilevel diabetes prevention interventions. In line with scoping review methodology, this review did not include a critical appraisal of the evidence, which would be a considerable challenge given that the heterogeneity in study designs limits the comparability of studies. In addition, the absence of a quality critical appraisal, which would be a challenge, given the variability of study designs, poses limitations for interpreting results. While our search strategy focused on diabetes-specific interventions, we may have missed interventions targeting relevant diabetes risk factors (eg, physical activity and BMI) if the article did not explicitly mention diabetes. Nonetheless, this review has identified important evidence gaps in equity outcomes reported in multilevel diabetes prevention interventions, which suggests directions for future research. In addition, our review focused on multilevel diabetes prevention interventions in high-income economies; therefore, the results may not be generalizable in low- and middle-income countries. Our review was also limited to only English articles, which may have introduced selection bias. The effectiveness of multilevel diabetes prevention interventions implemented in low- and middle-income economies, particularly in countries with pronounced diabetes inequities, can be the focus of future research.

### Conclusions

Our review demonstrated that multilevel diabetes prevention interventions have the potential to address population inequities in diabetes risk, given that these are often targeted for disadvantaged at-risk populations and are designed to take action on meso and macro-level health determinants. However, our review highlights that evidence of the impact of these interventions on reducing population inequities in diabetes incidence has not been examined or reported consistently. The findings from this review highlight the need for future studies to more explicitly address equity considerations in evaluations of multilevel diabetes prevention interventions. The “sick population” paradigm reminds us that focusing on individuals alone may overlook broader social and environmental factors [77]. Addressing diabetes inequities requires interventions that are responsive to community and systemic contexts, considering the distribution of risk within populations and accounting for health determinants across multiple levels of influence. Adopting a precision diabetes prevention approach may facilitate the implementation of interventions that are better aligned with community-specific needs, thereby increasing their relevance and potential impact on reducing inequities in diabetes risk.

## Supplementary material

10.2196/70267 Multimedia Appendix 1 Conceptual framework to define multilevel interventions and examples of health determinants relevant to diabetes risk.

10.2196/70267 Multimedia Appendix 2 Electronic database and gray literature search strategy.

10.2196/70267 Multimedia Appendix 3 Summaries of multilevel diabetes prevention interventions excluded from this scoping review due to an existing review.

10.2196/70267 Multimedia Appendix 4 Inclusion and exclusion criteria.

10.2196/70267 Multimedia Appendix 5 Study characteristics of articles included in this scoping review and description of multilevel diabetes prevention interventions, intervention coverage, and target populations.

10.2196/70267 Multimedia Appendix 6 Description of measured outcomes in studies reporting multilevel diabetes prevention interventions included in this scoping review (Extended).

10.2196/70267 Checklist 1 PRISMA-SCR checklist.

## Abbreviations

FI: food insecurity
PRESS: Peer Review for Electronic Search Strategies
PRISMA-ScR: Preferred Reporting Items for Systematic reviews and Meta-Analyses extension for Scoping Reviews
SES: socioeconomic status
