# Supplementary material for: Multilevel Diabetes Prevention Interventions to Address Population Inequities in Diabetes Risk: Scoping Review
Source: JMIR Public Health Surveill. 2025 Aug 25;11:e70267. doi: 10.2196/70267 (PMC12377877; doi:10.2196/70267)
Supplement: Multimedia Appendix 1 [file publichealth-v11-e70267-s001.docx]

**Multimedia Appendix 1: Conceptual framework to define multi-level interventions and examples of health determinants relevant to diabetes risk.**

| **Level of Influence** | | **Health Determinants** |
| --- | --- | --- |
| **Macro** | Socioeconomic Conditions, Environmental Factors, Structural Factors, Policy | • Income inequality  • Low education  • Low employment grade  • Built environment (e.g., urban spaces, active transport)  • Housing  • Food environment (food security, food access, food availability)  • Race and ethnicity  • Migration |
| **Meso** | Community Context | • Social network  • Community Connectedness  • Social Capital |
|  | Organizational / Institutional Settings | • Work environment  • School setting  • Health care / preventative services access  • Social Institutions |
| **Micro** | Interpersonal | • Social supports |
|  | Intrapersonal | • Lifestyle factors (diet, physical activity)  • Biological (sex, blood pressure, BMI) |
