# Supplementary material for: Multilevel Diabetes Prevention Interventions to Address Population Inequities in Diabetes Risk: Scoping Review
Source: JMIR Public Health Surveill. 2025 Aug 25;11:e70267. doi: 10.2196/70267 (PMC12377877; doi:10.2196/70267)
Supplement: Multimedia Appendix 2 [file publichealth-v11-e70267-s002.docx]

**Multimedia Appendix 2: Electronic database and grey literature search strategy**

Finalized search strategy (applied to Medline)

**1.** **exp Diabetes Mellitus, Type 2/**

**2.    exp hyperglycemia/**

**3.    prediabetic state/**

**4.    insulin resistance/**

**5.    (diabet* or diabetes mellitus type 2 or type 2 diabet* or type-2 diabet* or type II diabet* or adult-onset diabet* or non-insulin-dependent diabet* or hyperglycemia or high blood sugar or insulin resistance or insulin sensitivity or prediabet* or pre-diabet* or hyperglycemi*).mp.**

**6.    Lines 1 OR 2 OR 3 OR 4 OR 5**

**7.    Multilevel analysis/**

**8.    (Multilevel OR multi-level OR multiple level OR multicomponent OR multi-component OR multifaceted OR multi-faceted).mp.**

**9.    Lines 7 OR 8**

**10.    exp Preventative medicine/**

**11.    exp Preventive health services/**

**12.    Program evaluation/**

**13.    Policy/**

**14.    (prevent* OR preventive health services OR program* OR program evaluation OR policy OR intervention*).mp**

**15.   Lines 10 OR 11 OR 12 OR 13 OR 14**

**16.   Lines 6 AND 9 AND 15**

**17.   Limit 16: 2000-2024**

| **Website source** | **Search strategy** |
| --- | --- |
| **Centers for Disease Control and Prevention** | (diabet* OR “diabetes mellitus” AND “type 2 diabet*” OR “type ii diabet*” OR “adult-onset diabet*” OR “non-insulin-dependent diabet*” OR hyperglycemia OR “high blood sugar” OR “insulin resistance” OR “insulin sensitivity” OR prediabet* OR pre-diabet* OR hyperglycemi* ) AND (“multilevel intervention” OR “multi-level intervention” OR “multiple level intervention”) AND (prevent* OR “preventive health services” OR program* OR “program evaluation” OR policy) |
| **Government of Canda (covering Public Health Agency of Canada and Health Canada)** | (diabet* OR “diabetes mellitus” AND “type 2 diabet*” OR “type ii diabet*” OR “adult-onset diabet*” OR “non-insulin-dependent diabet*” OR hyperglycemia OR “high blood sugar” OR “insulin resistance” OR “insulin sensitivity” OR prediabet* OR pre-diabet* OR hyperglycemi* ) AND (“multilevel intervention” OR “multi-level intervention” OR “multiple level intervention”) AND (prevent* OR “preventive health services” OR program* OR “program evaluation” OR policy) |
| **Peel Public Health** | Website search |
| **Public Health Ontario** | Website search: diabetes prevention intervention (filter reports) |
| **Organization for Cooperation and Development** | Website search |
| **World Health Organization for Cooperation and Development** | (diabet* OR “diabetes mellitus” AND “type 2 diabet*” OR “type ii diabet*” OR “adult-onset diabet*” OR “non-insulin-dependent diabet*” OR hyperglycemia OR “high blood sugar” OR “insulin resistance” OR “insulin sensitivity” OR prediabet* OR pre-diabet* OR hyperglycemi* ) AND (“multilevel intervention” OR “multi-level intervention” OR “multiple level intervention”) AND (prevent* OR “preventive health services” OR program* OR “program evaluation” OR policy) |
