# Supplementary material for: Multilevel Diabetes Prevention Interventions to Address Population Inequities in Diabetes Risk: Scoping Review
Source: JMIR Public Health Surveill. 2025 Aug 25;11:e70267. doi: 10.2196/70267 (PMC12377877; doi:10.2196/70267)
Supplement: Multimedia Appendix 3 [file publichealth-v11-e70267-s003.docx]

**Multimedia Appendix 3: Summaries of multi-level diabetes prevention interventions excluded from this scoping review due to an existing review.**

| Review | Summary |
| --- | --- |
| Stotz, S.A., et al., Multi-level diabetes prevention and treatment interventions for Native people in the USA and Canada: a scoping review. Current Diabetes Reports, 2021. 21: p. 1-17. [1] | **Review aim:** This scoping review addresses the primary question: What is known in the existing literature about multi-level diabetes prevention interventions for Native peoples living in the United States and Canada?  **Type of Intervention:** Multi-level diabetes prevention interventions for Native Peoples  **Target Population:** Native Peoples  **Levels of Influence, Types of Health Determinants and Equity Factors Addressed:** The existing review found that multi-level interventions for Native Peoples generally address factors at the micro-, meso- and macro levels. These types of interventions have focused on addressing broader social determinants of health, such as access to healthy foods, safe spaces for physical activity, and socioeconomic factors.   **Outcomes Reported:** Physical activity, diet, blood pressure, weight/BMI  **Discussion:** Multi-level intervention design elements were largely individual-, school-, and community-based. Only three interventions included environmental or policy-level components. It was noted that involving members of these communities in the design and implementation of interventions ensures that the programs are relevant and effective, building trust and addressing the specific needs of the community. |
| Wnuk, K., et al., Workplace Interventions for Type 2 Diabetes Mellitus Prevention—an Umbrella Review. Current Diabetes Reports, 2023. 23(10): p. 293-304. [2] | **Review aim:** The purpose of this study is to identify and evaluate the effectiveness of workplace interventions aimed at preventing type 2 diabetes.  **Type of Intervention:** All interventions in the workplace aimed at diabetes prevention. Majority of interventions focused on workplace interventions based on guidelines of the US Diabetes Prevention Program or other similar programs.  **Target Population:** General population including both healthy people and diabetes risk-groups (including people with obesity).  **Levels of Influence, Types of Health Determinants and Equity Factors Addressed:** Interventions were multi-level, multi-component programs occurring within the workplace (meso-level) and including elements such as educational activities, interventions directed at changing diet, increasing physical activity, and reducing blood glucose and HbA1c levels (micro-level).  **Outcomes Reported:** Blood glucose level, glycated hemoglobin level, body weight, BMI and other parameters directly or indirectly indicating risks of diabetes.  **Discussion:** Authors concluded that an effective workplace intervention aimed to reduce the risk of type 2 diabetes among employees is a multicomponent program consisting of elements such as educational activities, interventions targeting dietary changes and increased physical activity. |
| Peñalvo, J.L., et al., Effectiveness of workplace wellness programmes for dietary habits, overweight, and cardiometabolic health: a systematic review and meta-analysis. The Lancet Public Health, 2021. 6(9): p. e648-e660. [3] | **Review aim:** The purpose of this systematic review and meta-analysis was to comprehensively study the effectiveness of multicomponent worksite wellness programmes for improving diet and cardiometabolic risk factors.  **Type of Intervention:** Eligible studies assessed multicomponent workplace wellness programs based on two or more intervention components that targeted improved health, such as the use of educational messages, cafeteria or vending machine interventions, promotion of stair use, financial incentives, changes to health insurance policies, or improved accessibility or discounts for gym memberships.  **Target Population:** Employees participating in workplace wellness programs.  **Levels of Influence, Types of Health Determinants and Equity Factors Addressed:** This study evaluated the impact across multiple levels of influence, including individual behaviours, social networks, and organizational policies. It also considered various health determinants such as biological, behavioural, social, and environmental factors.  **Outcomes Reported:** Change in dietary habits (measured by food frequency questionnaires, 24-hour recall, or dietary records), markers of adiposity (e.g., bodyweight, body-mass index [BMI], waist circumference, skinfolds, body fat percentage), cardiometabolic risk factors (e.g., blood pressure, lipids, glucose, insulin), cardiovascular risk scores (e.g., Framingham risk score), or disease outcomes (e.g., diabetes) if available.  **Discussion:** Authors concluded that workplace wellness programmes can improve specific dietary, anthropometric, and cardiometabolic risk indicators. |
| Mulchandani, R., et al., Effect of workplace physical activity interventions on the cardio-metabolic health of working adults: systematic review and meta-analysis. International Journal of Behavioral Nutrition and Physical Activity, 2019. 16: p. 1-16. [4] | **Review aim:** The aim of this systematic review and meta-analysis was to synthesize evidence for the effect of workplace physical activity interventions on the cardio-metabolic health markers among working adults.  **Type of Intervention:** Workplace studies implementing physical activity based interventions targeting inactivity to improve the cardio-metabolic disease markers (anthropometric and biochemical) in adult employees.  **Target Population:** Studies involving individuals aged 18 and above; healthy populations as well as populations at risk of CVD were included.  **Levels of Influence, Types of Health Determinants and Equity Factors Addressed:** This review included studies that involved multi-level interventions such as behavioural and social approaches (micro-level), and environmental and policy approaches (macro-level).   **Outcomes Reported:** Studies reporting any of the CVD outcomes (body weight, body fat, waist circumference, BMI, blood pressure, plasma glucose, lipids and triglycerides).  **Discussion:** Authors concluded that worksite physical activity interventions were effective at improving body weight, BMI and waist circumference of working adults. I |
| Roberts, S., et al., Efficacy of population‐wide diabetes and obesity prevention programs: an overview of systematic reviews on proximal, intermediate, and distal outcomes and a meta‐analysis of impact on BMI. Obesity Reviews, 2019. 20(7): p. 947-963. [5] | **Review aim:** The aim of the review was to assess the effectiveness of population-wide diabetes and obesity prevention programs by summarizing evidence from systematic reviews on various health outcomes, including behavioural changes, physiological markers, and disease incidence. Additionally, the review aimed to perform a meta-analysis to evaluate the impact of these programs specifically on BMI.  **Type of Intervention:** Population‐wide multi-level interventions aimed at preventing Type 2 diabetes mellitus or obesity in adults.  **Target Population:** General population.  **Levels of Influence, Types of Health Determinants and Equity Factors Addressed:** The interventions included in this review addressed multiple levels of influence, including individual behaviours, social networks, organizational policies, community initiatives, and broader health policies. It considered various health determinants such as biological, behavioural, environmental, social, and economic factors.  **Outcomes Reported:** Proximal outcomes of behaviour change (e.g., dietary habits, physical activity) and short-term physiological markers (e.g., blood glucose levels, cholesterol levels); intermediate outcomes that occur as a result of proximal changes but are more sustained, such as improvements in BMI, blood pressure, and other metabolic indicators; and, distal outcomes including the incidence of diabetes, obesity-related complications, and overall reductions in morbidity and mortality related to these conditions.  **Discussion:** Review identified a suite of population-wide actions to improve diet, increase physical activity, and reduce BMI. There was insufficient evidence of impact of any interventions on the prevalence of overweight, obesity, or Type 2 diabetes mellitus. |
| Virgara, R., et al., Interventions in outside-school hours childcare settings for promoting physical activity amongst schoolchildren aged 4 to 12 years. Cochrane Database Syst Rev, 2021. 9(9): p. Cd013380. [6] | **Review aim:** This review assessed the effectiveness, cost-effectiveness and associated adverse events of interventions designed to increase physical activity in children aged 4-12 years in outside-school hours childcare settings.  **Type of Intervention:** The intervention of interest was any program delivered in the outside‐school hours childcare setting (i.e. consistent childcare programs provided in the hours before or after school or during the school holiday period, or a combination of these) that aims to increase physical activity.  **Target Population:** Participants were primary/elementary school‐aged children attending outside‐school hours childcare services, where the children were predominantly aged 4 to 12 years.  **Levels of Influence, Types of Health Determinants and Equity Factors Addressed:** These interventions primarily focused on increasing physical activity through behaviour modification, environmental changes, and staff training. Equity factors included considering the socioeconomic context.    **Outcomes Reported:** The study measured primary outcomes such as total daily moderate-to-vigorous physical activity (MVPA) using various methods, and secondary outcomes including the proportion of care sessions spent in MVPA, cardiovascular health indicators like BMI and blood pressure, as well as evaluation outcomes like process implementation, cost-effectiveness, feasibility, adverse effects, and quality of life based on validated tools.  **Discussion:** Collectively, the findings suggest that interventions did not substantially improve primary or secondary outcomes in this setting, and this is likely due to the variation in intervention methods among included trials. In those studies that showed improvements in physical activity, multi‐component interventions had better results. These multi‐component interventions consisted of a physical activity guideline against which services could aim for and measure physical activity in the setting. In addition, they provided initial and ongoing staff training, which appeared to provide ongoing effects. Staff‐led sessions were also a component of successful interventions. |
| Hegarty, L. M., Mair, J. L., Kirby, K., Murtagh, E., & Murphy, M. H. (2016). School-based interventions to reduce sedentary behaviour in children: a systematic review. *AIMS public health*, *3*(3), 520. [7] | **Review Aim:** To conduct a systematic review to evaluate the effectiveness of school-based interventions designed to reduce sedentary behaviour on objectively measured sedentary time in children.  **Type of Intervention: I**nterventions aimed at reducing sedentary behaviour which involved the children's classroom.  **Target Population:** School-aged children.  **Levels of Influence, Types of Health Determinants and Equity Factors Addressed:** The review addressed multiple levels of influence: individual (behaviour change), interpersonal (teacher and peer support), and organizational (policy changes like standing desks). The interventions focused on health determinants such as behaviour modification and environmental changes. Equity factors were considered by designing interventions to be accessible and inclusive.  **Outcomes Reported:** Changes in sedentary time, physical activity levels, BMI and cardiovascular fitness.  **Discussion:** Authors concluded that multicomponent interventions which also include the use of standing desks may have a small effect for reducing children’s sedentary time in the school setting. There was no evidence of longitudinal effects. |
| Flórez, K.R., et al., Church-based interventions to address obesity among African Americans and Latinos in the United States: a systematic review. Nutrition reviews, 2020. 78(4): p. 304-322. [8] | **Review aim:** This systematic literature review of church-based interventions was conducted to assess their efficacy for addressing obesity across different racial/ethnic groups (eg, African Americans, Latinos).  **Type of Intervention:** Congregation-based obesity interventions in the United States.  **Target Population:** African Americans and Latinos  **Levels of Influence, Types of Health Determinants and Equity Factors Addressed:** The interventions included in the review addressed micro- and meso-levels of influence including individual behaviour changes and community engagement and social support. The interventions tackled various health determinants and equity factors such as socioeconomic barriers, cultural relevance, environmental access to healthy foods and safe physical activity spaces, and social support networks.  **Outcomes Reported:** Weight/waist circumference/BMI, dietary habits, physical activity, health knowledge, community engagement, cultural acceptability, and sustained behaviour changes.  **Discussion:** Authors concluded that by integrating faith-based elements and community involvement, these interventions effectively promote healthier behaviours. However, church-based interventions to address obesity will have greater impact if they are tailored to diverse populations (e.g., men of color, Latinos). |

**References**

1. Stotz SA, McNealy K, Begay RL, DeSanto K, Manson SM, Moore KR. Multi-level Diabetes Prevention and Treatment Interventions for Native People in the USA and Canada: a Scoping Review. Curr Diab Rep. 2021;21(11):46. doi:10.1007/s11892-021-01414-3

2. Wnuk K, Świtalski J, Tatara T, et al. Workplace Interventions for Type 2 Diabetes Mellitus Prevention—an Umbrella Review. Curr Diab Rep. 2023;23(10):293-304. doi:10.1007/s11892-023-01521-3

3. Peñalvo JL, Sagastume D, Mertens E, et al. Effectiveness of workplace wellness programmes for dietary habits, overweight, and cardiometabolic health: a systematic review and meta-analysis. Lancet Public Health. 2021;6(9):e648-e660. doi:10.1016/S2468-2667(21)00140-7

4. Mulchandani R, Chandrasekaran AM, Shivashankar R, et al. Effect of workplace physical activity interventions on the cardio-metabolic health of working adults: systematic review and meta-analysis. Int J Behav Nutr Phys Act. 2019;16(1):134. doi:10.1186/s12966-019-0896-0

5. Roberts S, Pilard L, Chen J, Hirst J, Rutter H, Greenhalgh T. Efficacy of population‐wide diabetes and obesity prevention programs: An overview of systematic reviews on proximal, intermediate, and distal outcomes and a meta‐analysis of impact on BMI. Obes Rev. 2019;20(7):947-963. doi:10.1111/obr.12821

6. Virgara R, Phillips A, Lewis LK, et al. Interventions in outside-school hours childcare settings for promoting physical activity amongst schoolchildren aged 4 to 12 years. Cochrane Public Health Group, ed. Cochrane Database Syst Rev. 2021;2021(9). doi:10.1002/14651858.CD013380.pub2

7. M. Hegarty L, L. Mair J, Kirby K, Murtagh E, H. Murphy M, 1 School of Sport, Ulster University, Jordanstown, Northern Ireland. School-based Interventions to Reduce Sedentary Behaviour in Children: A Systematic Review. AIMS Public Health. 2016;3(3):520-541. doi:10.3934/publichealth.2016.3.520

8. Flórez KR, Payán DD, Palar K, Williams MV, Katic B, Derose KP. Church-based interventions to address obesity among African Americans and Latinos in the United States: a systematic review. Nutr Rev. 2020;78(4):304-322. doi:10.1093/nutrit/nuz046
