# Supplementary material for: Multilevel Diabetes Prevention Interventions to Address Population Inequities in Diabetes Risk: Scoping Review
Source: JMIR Public Health Surveill. 2025 Aug 25;11:e70267. doi: 10.2196/70267 (PMC12377877; doi:10.2196/70267)
Supplement: Multimedia Appendix 4 [file publichealth-v11-e70267-s004.docx]

**Multimedia Appendix 4: Inclusion and exclusion criteria.**

| **Inclusion** | **Exclusion** |
| --- | --- |
| **Publication Type** | |
| Peer reviewed articles, grey literature indexed in web sources | Letters, editorials, opinion pieces, book chapters, conference abstracts |
| **Time frame** | |
| 2000-2024 | Before 2000 |
| **Geography** | |
| High income economies defined by World Bank Country and Lending Groups | low-income economies, lower-middle-income economies, upper-middle-income economies defined by World Bank Country and Lending Groups |
| **Language** | |
| English | Non-English |
| **Population/target group** | |
| Individuals without diabetes (youth and adults) | Individuals with diabetes (Type 1, Type 2, or gestational) |
| **Intervention** | |
| Multilevel diabetes prevention intervention aimed to target diabetes-relevant risk factors at more than one level (micro, meso, and macro) | - Interventions unrelated to diabetes prevention  -Interventions aimed to target diabetes-relevant risk factors at only one level (micro, meso, or macro)  - Multi-level diabetes prevention interventions for Native and Indigenous Peoples  -Diabetes prevention interventions implemented at the workplace setting  - built environment interventions related to investment in park/recreational areas, point-of decision prompts to encourage stair use, and active travel interventions  - food environment interventions related to economic measures (sugar-sweetened beverage prices, fast food prices, fruit and vegetable prices) and policy measures for food labelling or changing the layout/food provided at grocery stores  - interventions for physical activity in after school hours childcare settings (e.g., timetabling changes, provision of equipment for active play, changes in policies at regional or national level)  - school-based interventions aimed at reducing sedentary behaviour (e.g., changes to classroom design, such as sit-to-stand desks, changes to curriculum)  - church-based interventions targeted for African American and Latino communities |
| **Outcomes** | |
| Intervention outcomes reported (i.e., change in risk factors for diabetes (physical activity, BMI, diet, blood pressure), changes in diabetes risk, and changes in population inequities in diabetes risk) | No outcomes reported (e.g., description of intervention development or implementation) |
