# Supplementary material for: Multilevel Diabetes Prevention Interventions to Address Population Inequities in Diabetes Risk: Scoping Review
Source: JMIR Public Health Surveill. 2025 Aug 25;11:e70267. doi: 10.2196/70267 (PMC12377877; doi:10.2196/70267)
Supplement: Multimedia Appendix 5 [file publichealth-v11-e70267-s005.docx]

**Multimedia Appendix 5: Study characteristics of articles included in this scoping review and description of multi-level diabetes prevention interventions, intervention coverage, and target populations**

| Lead Author & Year of Publication | Study Country | Study design | Target population | Intervention coverage | Intervention |
| --- | --- | --- | --- | --- | --- |
| Mudd-Martin, G. (2013) [1] | United States | Community case study | Latinos | Not reported | Su Corazón, Su Vida is a community peer-worker (i.e., promotores) -facilitated educational program to reduce CVD risk among Latinos through healthy lifestyle promotion that has been successfully adapted for Type 2 diabetes prevention. Program consisted of 8 educational sessions and a ninth review/evaluation session. |
| Shin, A. (2015) [2] | United States | RCT | Low-income African American youth | Authors created an exposure score reflecting the amount of exposure to intervention activities. Total exposure score ranged from 0-6.74. Exposure in intervention was 2.02, compared to 0.98 in control. | The Baltimore Healthy Eating Zones intervention is a multi-level nutrition intervention introduced in recreation centres, corner stores, and/or carry-out restaurants. Activities were identified through formative work with community and consisted of the following components carried out in several phases: taste tests, cooking demonstrations, promoted foods (e.g., low-fat snacks, whole wheat bread, etc.), promoted behaviours (e.g., add vegetables into meals, choose baked over fried, etc.), educational activities, giveaways, shelf labels, and point-of-purchase health communication materials (posters + flyers). |
| Solomon, E. (2014) [3] | United Kingdom | RCT | Community (rural) | In intervention villages, 2.7% of the adult population (17+ years) registered to participate in at least one Devon Active Villages event. coverage was 5.2% among total population, including children. | Devon Active Villages is a community-level physical activity intervention coordinated by Active Devon, the Devon County partnership for sport and physical activity. Examples of intervention activities include basketball for primary school children, multi-sports sessions for adolescents, and fitness classes for adults. Intervention provided villages with 12-weeks of physical activity opportunities for all age groups, including at least three different types of activities per village. Each village received an individually-tailored intervention, incorporating a local needs-led approach. Support was provided to support activities for 12-months following the intervention. |
| Phillips, G. (2014) [4] | United Kingdom | RCT | Community (deprived, low-income) | The rate of participation in any Well London project measured by respondent recall in the follow-up survey was 3.1%. | The Well London is a public health intervention using community engagement and community-based projects to increase physical activity, healthy eating, mental health and well-being in London. Intervention comprised 14 interlinked projects, some of which focused on: Type 2 diabetes risk factors, improving local environments (e.g., green spaces), providing arts and cultural activities, and providing employment and training opportunities to residents.  At the beginning of the program, there was an extensive community consultation and engagement process in each neighbourhood to identify community needs and tailor the suite of projects. |
| Kloek, G. C. (2006) [5] | Netherlands | Quasi-experiment | Community (deprived, adult residents) | 43 activities were implemented. Participation ranged from <10 participants (14%); 11-25 participants (31%); 26-50 participants (12%); 51-100 participants (7%), and >100 participants (31%) | A community intervention to improve health-related behaviour among adults living in deprived neighbourhoods. Activities targeted health improvement and community empowerment goals. Intervention included: nutrition projects in primary schools, information on healthy nutrition and lifestyle for adults, collaboration with the greengrocer’s shop, neighbourhood walking tours, gymnastic classes, quit smoking courses, annual large community events related to health, and a monthly mailed newsletters. |
| De Cocker, K. A. (2007) [6] | Belgium | Controlled pre-post design | Community (population, 228,000) | 54% of participants reported hearing/seeing any information about physical activity promotion | Physical activity was promoted to all adults in the defined Ghent community, using a central theme of 10,000 steps/day, with a secondary tagline of ‘Every step counts.’ Strategies included a local media campaign; sale/loan of pedometers; dissemination of information through all associations, health professionals, schools; workplace projects; projects for older people; environmental approaches (street signs and walking circuits to encourage walking). |
| Brownson, R. C. (2005) [7] | United States | Quasi-experiment | Community (rural) | Exposure to intervention measured as a dose variable: low; medium; high (depending on attending an intervention event, residing in community with high media coverage on the intervention, reporting seeing promotion about the intervention, reporting that physician gave them exercise advice | Community-wide physical activity interventions developed with community input. Activities included: 1) developed walking trails in community with community input; 2) tailored newsletters and media events to promote walking; 3) interpersonal activities that stressed community social support (e.g., formed free walking groups) and health provider counseling at participating primary care offices (including emphasis on using the community walking trails); 4) community-wide events such as fun walks, trail events. |
| Brown, W. J. (2006) [8] | Australia | Quasi-experiment | Community (adult residents) | 70% recalled hearing messages about physical activity; 27% reported receiving physical activity advice from health professional; 18% reported using pedometer | Social marketing, healthcare provider, and environmental strategies were concurrently implemented with a central coordinating theme to increase physical activity. The use of pedometers as individual self-monitoring and goal-setting instruments was largely promoted by the media campaign. GPS and health professionals promoted physical activity and provided counselling. Policy and environmental change at local municipal government level led to improvements in built environment (e.g., creating and repairing footpaths, signs encouraging walking, distributing maps to encourage walking). |
| Richardson, A. (2017) [9] | United States | Quasi-experiment | Residents living in Low-income urban neighbour-hood experience-ing a food desert | Not reported | A full-service supermarket was opened in a low-income neighbourhood. The Healthy Food Financing Initiative incentivized supermarkets to locate in food deserts. The initiative was a partnership between the U.S. Departments of Treasury, Agriculture and Health and Human Services to provide financing in the form of tax credits, grants, or low-cost loans for developing and equipping grocery stores, small retailers, corner stores, and farmers markets selling healthy food in underserved areas. The grants were made as part of a broader strategy to address objectives like decreasing dependency on Federal programs, chronic unemployment, and community deterioration in rural areas. Conditions of the store and/or food prices were not regulated. |
| Joachim-Célestin , M. (2022) [10] | United States | Quasi-experiment | Adult, low-income, overweight and obese monolingual/bilingual (targeted to Spanish) Latinx women | Program retention was 79.6%; participation in different stages of the intervention ranged from 73.5%-100% | Full Plate Living is a culturally-adapted multi-component health intervention led by community health workers  using an education approach: 2-hour weekly sessions on preparing meals with food readily available at participants’ homes, shopping at favourite local stores, and eating out while on a small budget. Participants were encouraged to fill three-fourths of their plates with low-glycemic index fiber-rich foods (clearly colour-coded in a booklet) to create/complement favourite dishes; drinking water was available at each session and suggested as a replacement for juices/ sugar-sweetened beverages; and simple physical activity (mostly walking) was promoted. |
| Jago, R. (2011) [11] | United States | RCT | Community(middle school-aged children, grades 6-8) | 21 intervention schools (strategies for nutrition, physical activity, and marketing implemented as planned 84%-90% of time.  Schools were required to have at least 50% of students eligible for free or reduced-price lunch or belonging to an ethnic minority group, as well as having annual student dropout rate from aal causes ~25%. | The HEALTHY intervention had four components: 1) improved school food environment (improved nutritional quality), 2) physical activity and diet educational sessions, 3) social marketing, and 4) revised physical education curriculum. The focus of the intervention was on helping students to consume a healthier diet and engage in increased physical activity. |
| Wrigley, N. (2003) [12] | United Kingdom | Pretest-posttest design | Residents living in low-income and deprived community experience-ing a food desert | Of the 615 respondents completing both waves of the survey, 45% (276 respondents) reported switching to the new Tesco superstore as their `main' food retail source, with 35% (218) claiming the new store as their main fruit and vegetable source. | Intervention focused on inner city renewal through retail provision in the area aimed to improve food-retail access for healthy and affordable food in a deprived community experiencing a food desert. A retail area was redeveloped in the community by opening a large new superstore by UK's leading food retailer (Tesco) under a regeneration partnership with the local authority. This also supported employment, creating 230 new jobs in the community. |
| Cummins, S. (2005) [13] | United Kingdom | Quasi-experiment | Food-retail deficit community | Not reported | Provision of new large-scale food retailing in a food-retail deficit community. |
| Cochrane, T. (2008) [14] | United Kingdom | Quasi-experiment | Community (adult residents ≥16 years old, urban, materially deprived) | Intervention achieved respective participation targets for intervention activities (i.e., 1200 participants). At follow-up, 1275 individuals had participated in at least one activity. | 38 different types of activities were in introduced in accessible community areas in five broad categories: 1) walking (e.g., walking groups), 2) exercise referral, 3) sports, 4) water activities and 5) pastimes and active leisure pursuits. There was also a community awareness campaign. Intervention aimed to address the cultural norm of low physical activity by changing the environment and peer influences to promote health-enhancing physical activity within the community and increase the proportion of the community regularly engaging in physical activity by 10%. |
| Ashfield-Watt, PAL (2007) [15] | United Kingdom | Quasi experiment | Community (economic-ally deprived) | Not reported | In 2001 the UK Department of Health commissioned a pilot initiative to increase fruit and vegetable intake in five deprived communities by improving awareness, attitudes and access to fresh fruits and vegetables. These initiatives involved building community networks to achieve and sustain increased fruit and vegetable intakes through collaboration between retailers, educators, primary care teams, employers and local media. |
| McCurley, J. (2017) [16] | United States | 1-group pretest-posttest design | Latina women in mid-life (aged 45-65 years old) | Program enrollment rate was 58% among eligible women | Peer-led cultural adaptation of the original Diabetes Prevention Program lifestyle curriculum that emphasized health and lifestyle issues relevant to midlife Latina women (Nuestra Vida) and women with a history of gestational diabetes (Dulce Mothers). Group classes were held in community settings and were led by bilingual/bicultural Latina peer health educators (i.e., promotores). The adaptation retained core elements of the Diabetes Prevention Program curriculum (i.e., education on diabetes risk, healthy eating and physical activity, self-monitoring, goal setting) and added educational content specific to midlife (e.g., weight gain, menopause /hormonal changes; Nuestra Vida) or motherhood (e.g., breast-feeding, family health; Dulce Mothers), covered Latino cultural elements (e.g., foods, ethnicity-related risk factors, cultural beliefs), and emphasized skills for coping with stress and negative emotions and inclusion of peers and family into lifestyle changes. |
| VanStappen, V. (2021) [17] | Belgium, Finland, Greece, Spain, Hungary and Bulgaria | RCT | Community(parents and primary school aged children, ages 4-7, low-income) | Attendance rates for parental counseling sessions was on average 42.9%; all participating children received the intervention | Feel4Diabete was a 2-year multilevel (school-, community- and family-based) intervention developed to prevent Type 2 diabetes in vulnerable (low income) areas by promoting a healthy lifestyle in European families with an increased diabetes risk. Intervention components include counselling sessions aiming to inform families on risk factors related to Type 2 diabetes and to encourage them to adopt a healthier lifestyle (family level); motivational guidance via text messages (family level); information session for schoolteachers and headmasters on creating supportive social and physical environments (school level); and newsletters informing families about available infrastructure and existing health-related activities in the neighbourhood (community level). |
| Torrence, C. (2018) [18] | United States | Evaluation | Community(residents of low-income housing complexes, church participants living in low-income neighbour-hood; designated as low access food area) | coverage: 18 churches and four apartment complexes in low-income areas; 410 individuals completed the intervention. | Faithful Families Cooking and Eating Smart is a family-centred multi-level ecological intervention, created to improve nutrition and physical activity habits. The intervention included: nutrition and physical activity education component (nine nutrition and physical activity lessons that were designed to be facilitated by lay leaders from within faith organizations, with an evidence-based program, Cooking Matters, which empowers participants with the skills, knowledge, and confidence to make healthy and affordable meals); mobile farmers market (community and organizational change component operated for 6-weeks and functioned in the same manner as a traditional ice-cream truck; however, it was stocked with local produce); technical assistance and resources to area faith-based organizations to address the organizational barrier to healthy eating and physical activity. |
| Aubrey-Bassler, K. (2019) [19] | Canada | RCT | Community (adults aged 40-65, rural, remote and disadvantaged settings) | Eligibility for risk modification actions ranged from 14.3% - 87.4% (e.g., hypertension control (19%); diet score improvement (51.3%); physical activity improvement (85.0%); overweight stabilization (87.4%)) | BETTER 2 (Building on Existing Tools to Improve Chronic Disease Prevention and Screening in Primary Care) was a patient-based intervention with a prevention practitioner (PP) who met with patients in practice settings. The intervention included BETTER trial tools and shared decision-making to create a tailored 'prevention prescription' tailored to the patient's risk. Intervention components included: a BETTER health survey, a 60-minute clinical consultation about chronic disease prevention and screening with PP, shared-decision making with PP for patient goal-setting for prevention and screening, and follow-up visits with PP. |
| Simon, C. (2008) [20] | France | RCT | Adolescents (6^th^ graders) | All the six-graders of the intervention schools were exposed to the program. On average, 90% of the study participants attended the educational classes and debates. Regular participation in physical activities offered in the program, defined as a mean participation time of at least 30 mins/week, increased from 25% in the first months to 65% during the last 2 years of follow-up. | The Intervention Centred on Adolescents is a multi-level intervention that integrates environmental changes to reduce the barriers to adopting an active lifestyle. The program included: an educational component focusing on physical activity and sedentary behaviours; new opportunities for physical activity at lunchtime, during breaks and afterschool hours; sporting events and 'cycling to school' days. The objectives were to change attitudes toward physical activity; promote social support by parents/educators; provide environmental and institutional conditions to encourage physical activity. |
| Novotny, R. (2018) [21] | United States | RCT | Children of US-affiliated Pacific Islanders (USAP) region communities | Not reported | The Children's Healthy Living program was a multi-jurisdictional, multi-level, multicomponent intervention consisting of 19 activities which were selected to address target behaviours derived from community-informed ideas. The activities were grouped into the following 4 strategies: organizational policy change, environmental change, social marketing, and training.  These strategies also addressed the interpersonal (training role models, parents, and teachers), community (increasing access to healthy foods and environments for safe play), and organizational and policy (strengthening preschool wellness policies) levels of the social-ecological model. |
| Ivester, P. (2010) [22] | United States | Quasi-experiment | Community(overweight and obese adults, attending church congregates) | 41 (out of 46) participants who began the program completed it: 89% | Multifaceted, church-based wellness program consisting of the following activities: 2 weekly educational programs (approx. 5 hours total); 5-component dietary recommendations: calorie reduction, increased fiber intake, increased polyphenol intake, increased intake of long-chain omega-3 fatty acids, increased intake of short-chain omega-6 fatty acids; exercise recommendations of at least 30 minutes/day: aerobic exercise 3 days/week and circuit training 2 days/week; Weekly meetings at church for: body measurements, group exercise sessions, dietary counselling, and group support. |
| Frediani, J. K. (2021) [23] | United States | One-arm pilot clinical trial | Hispanic men (overweight and obese, aged 30-55 years with prediabetes) | Average attendance was 65% | The intervention was an adaptation of the Centers for Disease Control and Prevention’s National Diabetes Prevention Program (NDPP) for Hispanic males through the incorporation of a recreational soccer (RS) component. Trained soccer coaches led 30-min facilitated discussion of the NDPP modules after each RS session, with two sessions per week for 12-weeks and once per week for the following 12-weeks. The program activities included: RS conditioning (evidence-based 15-minute dynamic warm-up, 20 minutes of soccer-specific drills with and without the ball, and 20 minutes of recreational short-sided soccer games 5v5 up to 7v7 format depending on session attendance); core curriculum modules; and a closed and encrypted chat (WhatsApp platform) was created for each cohort and for the full group to facilitate peer support, communication, and session attendance (i.e., schedule changes). |
| Chesla, C. A. (2016) [24] | United States | Cohort study | Chinese immigrants (adults, overweight, meeting criteria for prediabetes) | Participants attended on average 77% of the 16 sessions offered. | Chinese American Lifestyle Education program, which is a cultural adaptation of the federally funded Group Lifestyle Balance Program. It is a lifestyle change program which focuses on healthy eating and increasing physical activity. The program included core phase (12 weekly session, over 3 months); transition phase (4 sessions over 3 months). Goal was to achieve weight loss through healthy eating and weekly physical activity. |
| Andersen, E. (2012) [25] | Norway | RCT | Pakistani immigrant men (aged 25-60 years old, low physical activity) | Participation in different intervention components ranged from 60%-100% | Multi-component physical activity intervention was tailored to target population to promote physical activity change. The program included structured group exercise sessions led by an exercise physiologist twice a week, two group lectures, one individual counselling session, written material, and a phone call. |

**References**

1. Mudd-Martin G, Martinez MC, Rayens MK, Gokun Y, Meininger JC. Sociocultural Tailoring of a Healthy Lifestyle Intervention to Reduce Cardiovascular Disease and Type 2 Diabetes Risk Among Latinos. *Prev Chronic Dis*. 2013;10:130137. doi:10.5888/pcd10.130137

2. Shin A, Surkan PJ, Coutinho AJ, et al. Impact of Baltimore Healthy Eating Zones: An Environmental Intervention to Improve Diet Among African American Youth. *Health Educ Behav*. 2015;42(1_suppl):97S-105S. doi:10.1177/1090198115571362

3. Solomon E, Rees T, Ukoumunne OC, Metcalf B, Hillsdon M. The Devon Active Villages Evaluation (DAVE) trial of a community-level physical activity intervention in rural south-west England: a stepped wedge cluster randomised controlled trial. *Int J Behav Nutr Phys Act*. 2014;11(1):94. doi:10.1186/s12966-014-0094-z

4. Phillips G, Bottomley C, Schmidt E, et al. Measures of exposure to the *Well London* Phase-1 intervention and their association with health well-being and social outcomes. *J Epidemiol Community Health*. 2014;68(7):597-605. doi:10.1136/jech-2013-202507

5. Kloek GC, Van Lenthe FJ, Van Nierop PWM, Koelen MA, Mackenbach JP. Impact evaluation of a Dutch community intervention to improve health-related behaviour in deprived neighbourhoods. *Health Place*. 2006;12(4):665-677. doi:10.1016/j.healthplace.2005.09.002

6. De Cocker KA, De Bourdeaudhuij IM, Brown WJ, Cardon GM. Effects of “10,000 Steps Ghent.” *Am J Prev Med*. 2007;33(6):455-463. doi:10.1016/j.amepre.2007.07.037

7. Brownson RC, Hagood L, Lovegreen SL, et al. A multilevel ecological approach to promoting walking in rural communities. *Prev Med*. 2005;41(5-6):837-842. doi:10.1016/j.ypmed.2005.09.004

8. Brown WJ, Mummery K, Eakin E, Schofield G. 10,000 Steps Rockhampton: Evaluation of a Whole Community Approach to Improving Population Levels of Physical Activity. *J Phys Act Health*. 2006;3(1):1-14. doi:10.1123/jpah.3.1.1

9. Richardson AS, Ghosh-Dastidar M, Beckman R, et al. Can the introduction of a full-service supermarket in a food desert improve residents’ economic status and health? *Ann Epidemiol*. 2017;27(12):771-776. doi:10.1016/j.annepidem.2017.10.011

10. Joachim-Célestin M, Rockwood NJ, Clarke C, Montgomery SB. Evaluating the Full Plate Living lifestyle intervention in low-income monolingual Latinas with and without food insecurity. *Womens Health*. 2022;18:174550572210913. doi:10.1177/17455057221091350

11. Jago R, Mcmurray RG, Drews KL, et al. HEALTHY Intervention: Fitness, Physical Activity, and Metabolic Syndrome Results. *Med Sci Sports Exerc*. 2011;43(8):1513-1522. doi:10.1249/MSS.0b013e31820c9797

12. Wrigley N, Warm D, Margetts B. Deprivation, Diet, and Food-Retail Access: Findings from the Leeds ‘Food Deserts’ Study. *Environ Plan Econ Space*. 2003;35(1):151-188. doi:10.1068/a35150

13. Cummins S. Large scale food retailing as an intervention for diet and health: quasi-experimental evaluation of a natural experiment. *J Epidemiol Community Health*. 2005;59(12):1035-1040. doi:10.1136/jech.2004.029843

14. Cochrane T, Davey RC. Increasing uptake of physical activity: a social ecological approach. *J R Soc Promot Health*. 2008;128(1):31-40. doi:10.1177/1466424007085223

15. Ashfield-Watt P, Welch A, Godward S, Bingham S. Effect of a pilot community intervention on fruit and vegetable intakes: use of FACET (Five-a-day Community Evaluation Tool). *Public Health Nutr*. 2007;10(7):671-680. doi:10.1017/S1368980007382517

16. McCurley JL, Fortmann AL, Gutierrez AP, et al. Pilot Test of a Culturally Appropriate Diabetes Prevention Intervention for At-Risk Latina Women. *Diabetes Educ*. 2017;43(6):631-640. doi:10.1177/0145721717738020

17. Van Stappen V, Cardon G, De Craemer M, et al. The effect of a cluster-randomized controlled trial on lifestyle behaviors among families at risk for developing type 2 diabetes across Europe: the Feel4Diabetes-study. *Int J Behav Nutr Phys Act*. 2021;18(1):86. doi:10.1186/s12966-021-01153-4

18. Torrence C, Griffin SF, Rolke L, Kenison K, Marvin A. Faithful Families Cooking and Eating Smart and Moving for Health: Evaluation of a Community Driven Intervention. *Int J Environ Res Public Health*. 2018;15(9):1991. doi:10.3390/ijerph15091991

19. Aubrey-Bassler K, Fernandes C, Penney C, et al. The effectiveness of a proven chronic disease prevention and screening intervention in diverse and remote primary care settings: an implementation study on the BETTER 2 Program. *BJGP Open*. 2019;3(3):bjgpopen19X101656. doi:10.3399/bjgpopen19X101656

20. Simon C, Schweitzer B, Oujaa M, et al. Successful overweight prevention in adolescents by increasing physical activity: a 4-year randomized controlled intervention. *Int J Obes*.

21. Novotny R, Davis J, Butel J, et al. Effect of the Children’s Healthy Living Program on Young Child Overweight, Obesity, and Acanthosis Nigricans in the US-Affiliated Pacific Region: A Randomized Clinical Trial. *JAMA Netw Open*. 2018;1(6):e183896. doi:10.1001/jamanetworkopen.2018.3896

22. Ivester P, Sergeant S, Danhauer S, et al. Effect of a Multifaceted, Church-Based Wellness Program on Metabolic Syndrome in 41 Overweight or Obese Congregants. *Cent Dis Control Prev*. 2010;7(4):1-8.

23. Frediani JK, Li J, Bienvenida A, Higgins MK, Lobelo F. Metabolic Changes After a 24-Week Soccer-Based Adaptation of the Diabetes Prevention Program in Hispanic Males: A One-Arm Pilot Clinical Trial. *Front Sports Act Living*. 2021;3:757815. doi:10.3389/fspor.2021.757815

24. Chesla CA, Chun KM, Kwong Y, et al. Cultural Adaptation of the Group Lifestyle Balance Program for Chinese Americans. *Diabetes Educ*. 2016;42(6):686-696. doi:10.1177/0145721716666679

25. Andersen E, Burton NW, Anderssen SA. Physical activity levels six months after a randomised controlled physical activity intervention for Pakistani immigrant men living in Norway. *Int J Behav Nutr Phys Act*. 2012;9(1):47. doi:10.1186/1479-5868-9-47
