# Supplementary material for: Multilevel Diabetes Prevention Interventions to Address Population Inequities in Diabetes Risk: Scoping Review
Source: JMIR Public Health Surveill. 2025 Aug 25;11:e70267. doi: 10.2196/70267 (PMC12377877; doi:10.2196/70267)
Supplement: Multimedia Appendix 6 [file publichealth-v11-e70267-s006.docx]

**Multimedia Appendix 6: Description of measured outcomes in studies reporting multi-level diabetes prevention interventions included in this scoping review (Extended)**

| Lead Author | Intervention type | Group reported on | Outcomes reported | Measures |
| --- | --- | --- | --- | --- |
| Mudd-Martin, G. [1] | Targeted for high-risk | Population group (Latinx community) | Physical Activity   - Statistically significant increase in PA post-intervention (pre-intervention: 2.0, post-intervention: 2.4, p=0.003).   Diet   - No statistically significant differences in diet post-intervention (pre-intervention: 2.5 post-intervention: 2.7, p=0.20). | Health-Promoting Lifestyle Profile (HPLP-II) subscale scores for physical activity and diet |
| Shin, A. [2] | Environmental | Population group (low-income African American youth) | Weight/BMI   - Statistically significant decrease in BMI for age percentiles in the intervention group compared to control group (BMI age percentile pre-intervention: 73.6, post-intervention: 71.4, difference: -2.23, p=0.04). | Height and weight measurement |
| Solomon, E. [3] | Community | Total population | Physical Activity   - No statistically significant changes in meeting physical activity guidelines (p=0.8). Statistically significant increases in meeting minutes of exercise per week (p=0.07) and increases in PA habits (p=0.009). | International Physical Activity Questionnaire (IPAQ-SV) |
| Phillips, G. [4] | Community | Total population and population group (subgroups by ethnicity and education) | Physical Activity   - No statistically significant changes in 5x30 minute moderate intensity activity per week in total population (p=0.9) or subgroups by ethnicity and education.   Diet   - No statistically significant changes in healthy eating diet (meeting 5/day fruit and/or vegetables) (p=0.5). Statistically significant reductions in unhealthy eating score (p=0.03) in total population. No changes in subgroups by ethnicity and education. | General Health Questionnaire (GHQ) |
| Kloek, G. C. [5] | Community | Total population | Physical Activity   - No statistically significant changes in PA score (p=0.65).   Diet   - Compared to control group, the intervention group had statistically significant increases in fruit self-efficacy score (p=0.048) and fruit consumption (p=0.044), but not in vegetable consumption (p=0.12). | Short Questionnaire to  Assess Health enhancing PA (SQUASH) questionnaire  Food Frequency Questionnaire (FFQ) |
| De Cocker, K. A. [6] | Community | Total population | Physical Activity   - Significant increases in self-reported walking (min/week) (p<0.05) and pedometer steps/day (p=0.001). | International Physical Activity Questionnaire (IPAQ), Pedometer |
| Brownson, R. C. [7] | Community | Total population and population group (subgroups by ethnicity, household income, and education) | Physical Activity (total pop)   - The amount of change in walking/week at follow-up was higher in intervention (11.7 min) than in comparison participants (6.5 min), although not statistically significant (p=0.864).   Physical Activity (pop group)   - < High school education: There was a decrease in walking/week in intervention group compared to control group (-1.4 minutes), although not statistically significant. - < $20,000 household income: There was an overall increase in walking/week in intervention group compared to control group (+16.0 minutes), although not statistically significant. - African American group: There was a decrease in walking/week in intervention group compared to control group (-18.5 minutes), although not statistically significant. | Behavioral Risk Factor  Surveillance System, telephone survey |
| Brown, W. J. [8] | Environmental | Total population and population group (subgroup by sex) | Physical Activity (total pop)   - No statistically significant changes in walking scores in intervention group categorized as active at follow-up compared to baseline (+0.9% difference, OR=0.98).   Physical Activity (pop group)   - In women categorized as active, there was an increase in walking scores at follow-up compared to baseline, although not statistically significant (5% difference, OR=1.24). - In men categorized as active, there was a decrease in walking scores at follow-up compared to baseline, although not statistically significant (-4.2%, OR=0.83). | Pedometers |
| Richardson, A. [9] | Environmental | Total population | Metabolic risk   - Increase in high blood sugar in the intervention neighbourhood compared to control neighbourhood, although not statistically significant (p=0.18). - Decrease in self-reported diabetes in intervention neighbourhood compared to control neighbourhood, although not statistically significant (p=0.10).   Weight/BMI   - No change in mean BMI in intervention neighbourhood compared to control neighbourhood (p>0.05).   Diet   - No change in fruit and vegetable servings in intervention neighbourhood compared to control neighbourhood (p>0.05).   Blood pressure   - Significant increase in hypertension in both intervention neighbourhood and control neighbourhood. The difference in difference was not significant (relative change=0.5%, p=0.87). | Not available  Height and weight measurement  Automated self-administered 24-hour recall (ASA-24)  Self-reported hypertension |
| Joachim-Célestin, M. [10] | Targeted for high-risk | Population group (Latinx women) | Weight/BMI   - Statistically significant decrease in weight (lbs.) among overweight and obese Latinas in both food insecure group (p=.001 and p=.006) and food secure group (p=.006 and p=.02), 3 months post-intervention.   Physical Activity   - Statistically significant increase in physical activity levels (mins) among Latinas in both food insecure group (p=.01) and food secure group (p=.003), 3 months post-intervention.   Diet   - Statistically significant increase of fiber-rich food consumption at 3 months compared to baseline in both food insecure group (p=.03) and food secure group (p<.001). | Height and weight measurement  International Physical Activity Questionnaire  Self-reported surveys |
| Jago, R. [11] | Community | Total population and population group (subgroups by sex and ethnicity) | Metabolic risk   - Among the total study population, there were no statistically significant changes in metabolic syndrome prevalence (5.7% baseline, 5.1% 3-year follow-up, p=0.3443). - Among Hispanic, Black, and White male and female participants, there were no statistically significant changes in metabolic syndrome prevalence [+3.7% (male/Hispanic); +1.0% (male/Black); +16.0% (male/White); -1.6% (female/Hispanic); -3.5% (female/Black); -1.3% (female/White)].   Weight/BMI   - In overweight/obese students (BMI ≥ 85 percentile) om the intervention school, there was a decrease in the prevalence of students classified as BMI ≥ 85 percentile (-4.5%; 50.3% baseline, 45.8% post 3-year follow-up). There were no significant differences between the intervention and control schools (OR=0.99; p=0.92). - In obese students (BMI ≥ 95 percentile) in the intervention schools, there was a decrease in prevalence of BMI ≥ 95 percentile (-5.5%; 30.1% baseline, 24.6% post 3-year follow-up). There was a significant difference between the intervention and control schools (OR=0.81; p=0.05).   Physical Activity   - Among the intervention schools, there was a decrease in mean moderate-to-vigorous physical activity (MVPA) (minutes/day), although not statistically significant (-7.9 min; 101.2 min baseline, 93.3 min, 3-year post follow-up, p=0.2520). - Among Hispanic, Black, and White participants in the intervention school, there were no statistically significant changes in mean MVPA (minutes/day) [+4.3 min, p=0.30 (male/Hispanic); +0.8 min, p=1.13 (male/Black); +16.0 min, p=0.71 (male/White); -18.9 min, p=0.82 (female/Hispanic); -21.7 min, p=0.28 (female/Black); -6.1 min, p=0.61 (female/White)]. | Anthropometric measures  Height and weight measurement; BMI age-and gender-specific BMI percentiles  Self-Administered Physical Activity Checklist  (SAPAC) |
| Wrigley, N. [12] | Environmental | Total population and population group (defined by diet) | Diet (total pop)   - Among the total study population, there was an increase in fruit/vegetable consumption (portions/day), although not statistically significant (0.4; 2.88, baseline, 2.92 post-intervention, p=0.484).   Diet (pop group)   - Among the group with poor diet pre-intervention (≤2 portions/day) there was an increase in fruit/vegetable consumption (portions/day), although not statistically significant (0.44; 1.31, baseline, 1.75 post-intervention, p>0.05). - Among the group with worst diets pre-intervention (≤1 portions/day), there was an increase in fruit/veg consumption (portions/day), although not statistically significant (0.82; 0.58, baseline, 1.40 post-intervention, p>0.05). | National Food Survey (NFS) |
| Cummins, S. [13] | Environmental | Total population | Diet   - No statistically significant changes in fruit/vegetable consumption (portions/day) in the intervention community post-intervention compared to pre-intervention (mean change=0.09, p=0.07). | Self-reported fruit and vegetable consumption |
| Cochrane, T. [14] | Community | Total population | Physical Activity   - Statistically significant higher self-reported being more physically activity than 1 year prior in intervention community compared to control community (30.6%, intervention; 18.3%, control; p ≤ 0.001). | Self-reported change in physical activity |
| Ashfield-Watt, PAL [15] | Environmental | Total population | Diet   - No statistically significant change in fruit/vegetable consumption (portions/day) from baseline to follow-up in intervention group (mean change=0.1; p>0.05). | Five-a-day Community Evaluation Tool (FACET) |
| McCurley, J. [16] | Targeted for high-risk | Population group (Latinx women) | Metabolic risk   - No statistically significant change in A1C (%) (5.66%, baseline; 5.63%, 6-month follow-up; p=0.67).   Weight/BMI   - No significant decrease in weight (lb.) (169.4, baseline; 162.8, 6-month follow-up; p=0.28). - No significant decrease in BMI (mean) (31.6, baseline; 30.5, 6-month follow-up; p=0.27).   Physical Activity   - No statistically significant increase in physical activity aerobic score (mean) (4.4, baseline; 5.7, 6-month follow-up; p=0.08).   Diet   - Statistically significant increase in healthful eating scores (46.0, baseline; 51.3, 6-month follow-up; p=0.03).   Blood pressure   - No statistically significant change in systolic blood pressure (mmHg) (118.1, baseline; 118.6, 6-month follow-up; p=0.69) and diastolic blood pressure (mmHg) (71.8, baseline; 72.0, 6-month follow-up; p=0.67). | Siemens DCA Vantage  Analyzer  Height and weight measurement  9 item Rapid Assessment of Physical Activity  University of California Cooperative Extension Food Behavior Checklist  Automated Oscillo metric device (Omron  HEM 907XL) |
| VanStappen, V. [17] | Community | Total population and population group (subgroups by age) | Physical Activity (total pop)   - No statistically significant change in moderate-to-vigorous physical activity (MVPA) post-intervention among parents across all countries (F=2.42, p=0.09). - Statistically significant increase in MVPA post-intervention among children across all countries (F=3.37, p=0.03).   Diet (total pop)   - Statistically significant increase in water consumption (F=3.24, p=0.04) and fruit/vegetable consumption (F=3.31, p=0.04) post-intervention among parents across all countries. - No statistically significant changes in water consumption (F=0.28, p>0.75), fruit/vegetable (F=1.44, p=0.24), sugar-sweetened beverage (F=0.33, p=0.71), salty/fast food (F=2.89, p=0.06) and breakfast consumption (F=0.75, p=0.47) post-intervention among children across all countries. - No statistically significant change in sweets (F=1.51, p=0.22), salty/fast food (F=0.40, p=0.67), and breakfast consumption (F=1.24, p=0.29) post-intervention among parents across all countries. - Statistically significant decrease in sweets consumption (F=5.13, p=0.01) post-intervention among children across all countries. | Self-reported moderate-to-vigorous physical activity (MVPA)  Self-reported questions on diet |
| Torrence, C. [18] | Community | Total population | Physical Activity   - Statistically significant increase in mean reported change in physical activity frequency and intensity (baseline 4.8, post 6-week 5.3; p<0.01) and strength and flexibility score (baseline 1.0, post 6-week 1.8, p<0.001) among participants.   Diet   - No significant change in fruit consumption and vegetable consumption scores. Statistically significant increase in low fat dairy consumption score (baseline 3.0, post 6-week 3.3, p<0.01). | Rapid Assessment of Physical Activity Score  Cooking Matters assessment |
| Aubrey-Bassler, K. [19] | Community | Total population | Physical Activity   - Among participants eligible for physical activity improvement, 67.0% achieved improvement in physical activity from baseline.   Diet   - Among participants eligible for diet score improvement, 62.1% achieved a diet score improvement from baseline.   Blood pressure   - Among participants eligible for hypertension control improvement, 85.7% achieved hypertension control from baseline. | General Practice Physical Activity Questionnaire (GPPAQ)  Starting the Conversation dietary assessment tool  Physical examinations |
| Simon, C. [20] | Environmental | Population group (6^th^ graders) | Metabolic risk   - No significant change in mean plasma glucose (mg per 100 ml) in intervention group compared to control after 4-year follow-up (0.00, fourth year baseline difference; p=0.81).   Weight/BMI   - Significant decrease in gender-age adjusted BMI in initially non-overweight students in intervention group compared to control after 4-year follow-up (-0.36, fourth year baseline difference, p<0.01). - No significant change in gender-age adjusted BMI in initially overweight students in intervention group compared to control after 4-year follow-up (0.16, fourth year baseline difference, p=0.68).   Physical Activity   - Significant increase in mean leisure physical activity (hours/week) pressure in intervention group compared to control after 4-year follow-up (1.10, fourth year baseline difference, p<0.0001).   Blood pressure   - No significant change in systolic blood pressure and diastolic blood pressure in intervention group compared to control after 4-year follow-up (-0.42, fourth year baseline difference; p=0.66 and -0.46, fourth year baseline difference; p=0.60, respectively). | Fasting blood plasma glucose (laboratory measurement)  Height and weight measurement  Modifiable Activity Questionnaire for adolescents  Blood pressure measurement performed by qualified professional |
| Novotny, R. [21] | Targeted for high-risk | Population group (children of US-Affiliated Pacific Islanders) | Metabolic risk   - Statistically significant difference in decrease in acanthosis nigricans (skin indicator of insulin resistance) prevalence among children in the intervention community compared to control community (-2.58% vs. -0.30%, p<0.001).   Weight/BMI   - Statistically significant difference in decrease in overweight/obesity prevalence among children in intervention community compared to control community (-3.95%, p=0.02).   Physical Activity   - No statistically significant changes in moderate-to-vigorous physical activity (mins/day) (1.16, p=0.55) and accelerometer total METS (mins/day) (-0.11, p=0.38) among children in intervention community compared to control community.   Diet   - No statistically significant difference in fruit (cups/day) (-0.03, p=0.55) and vegetable (cups/day) (-0.004, p=0.90) consumption among children in the intervention community compared to control community. | Back of neck test using scale developed by Burke et al., 1999  Height and weight measurement  Omnidirectional accelerometers and questionnaire on screen-time  2 dietary records on randomly selected days |
| Ivester, P. [22] | Community | Total population | Metabolic risk   - Significant decrease in HbA1c % in women (6.0, baseline; 5.5, 8-week follow-up; p<0.001) and men (5.9, baseline; 5.7, 8-week follow-up; p<0.006).   Weight/BMI   - Statistically significant decrease in average BMI in women (32, baseline; 30, 8-week follow-up; p<0.05) and men (31, baseline; 29, 8-week follow-up; p<0.05).   Blood pressure   - Statistically significant decrease in systolic blood pressure for men (134.7, baseline; 127.1 8-week follow-up; p<0.05). No statistically significant change in systolic blood pressure for women (134.9, baseline; 130.5, 8-week follow-up; p>0.05) and diastolic blood pressure for both men (84.3, baseline; 81.2, 8-week follow-up; p>0.05) and women (80.2, baseline; 79.8, 8-week follow-up; p>0.05). | Fasting blood samples  Height and weight measurement; Tanita BF 350 body fat scale  Anthropometric measurement |
| Frediani, J. K. [23] | Targeted for high-risk | Population group (Hispanic males) | Metabolic risk   - No significant change in mean HbA1c (%) at 24-weeks among Hispanic males (5.7, baseline; 5.6, post 24-weeks, p=0.23).   Weight/BMI   - Statistically significant decrease in mean BMI (32.7 baseline, 31.4 post 24-weeks follow-up, p<0.05) among Hispanic males.   Diet   - No significant change in fruit intake (units) (2.2, baseline; 1.7 post 24-weeks; 95% CI -1.2, 0.2). No significant change in vegetable intake (units) (3.2, baseline; 3.1, post 24-weeks, 95% CI -0.9, 0.7).   Blood pressure   - Statistically significant decrease in diastolic blood pressure (140.6, baseline; 134.0, post 24-weeks, p<0.01) and systolic blood pressure (90.1, baseline; 84.0, post 24-weeks; p<0.01) among Hispanic males. | Anthropometric measures  Height and weight measurement  24-h dietary recall (validated multi-pass method)  Calibrated electronic blood pressure sphygmomanometer |
| Chesla, C. A. [24] | Targeted for high-risk | Population group (Chinese immigrants) | Metabolic risk   - No statistically significant change in fasting plasma glucose (mg/dl) (96.4, baseline; 93.1, post 6-months; p>0.05) among study participants. - No significant change in A1c % levels (5.91, baseline; 5.89, post 6-months; p>0.05) among study participants.   Weight/BMI   - Statistically significant decrease in BMI (29.4, baseline; 27.5, post 6-months; p<0.001) among study participants.   Physical Activity   - Statistically significant increase in self-reported physical activity weekly METS (829, baseline; 1640, post 6-months; p<0.01) among study participants.   Diet   - Statistically significant decrease in dietary intake Starting the Conversation (5.9, baseline; 5.1, post 6-months; p<0.01) among study participants.   Blood pressure   - Statistically significant decrease in diastolic blood pressure (82.2, baseline; 78.4, post 6-months; p<0.05) among study participants. | Blood tests  Height and weight measurement  International Physical Activity Questionnaire  the 8-item Starting the Conversation (STC) diet  score; 27-item Dietary Screener Questionnaire  Anthropometric measures |
| Andersen, E. [25] | Targeted for high-risk | Population group (Pakistani immigrant males) | Physical Activity   - Statistically significant difference in the increase in total physical activity level (counts per min/day) in the intervention group compared to control group at post 6-months (81 adjusted difference, p=0.001). | ActiGraph accelerometers |

References

1. Mudd-Martin G, Martinez MC, Rayens MK, Gokun Y, Meininger JC. Sociocultural Tailoring of a Healthy Lifestyle Intervention to Reduce Cardiovascular Disease and Type 2 Diabetes Risk Among Latinos. Prev Chronic Dis. 2013;10:130137. doi:10.5888/pcd10.130137

2. Shin A, Surkan PJ, Coutinho AJ, et al. Impact of Baltimore Healthy Eating Zones: An Environmental Intervention to Improve Diet Among African American Youth. Health Educ Behav. 2015;42(1_suppl):97S-105S. doi:10.1177/1090198115571362

3. Solomon E, Rees T, Ukoumunne OC, Metcalf B, Hillsdon M. The Devon Active Villages Evaluation (DAVE) trial of a community-level physical activity intervention in rural south-west England: a stepped wedge cluster randomised controlled trial. Int J Behav Nutr Phys Act. 2014;11(1):94. doi:10.1186/s12966-014-0094-z

4. Phillips G, Bottomley C, Schmidt E, et al. Measures of exposure to the Well London Phase-1 intervention and their association with health well-being and social outcomes. J Epidemiol Community Health. 2014;68(7):597-605. doi:10.1136/jech-2013-202507

5. Kloek GC, Van Lenthe FJ, Van Nierop PWM, Koelen MA, Mackenbach JP. Impact evaluation of a Dutch community intervention to improve health-related behaviour in deprived neighbourhoods. Health Place. 2006;12(4):665-677. doi:10.1016/j.healthplace.2005.09.002

6. De Cocker KA, De Bourdeaudhuij IM, Brown WJ, Cardon GM. Effects of “10,000 Steps Ghent.” Am J Prev Med. 2007;33(6):455-463. doi:10.1016/j.amepre.2007.07.037

7. Brownson RC, Hagood L, Lovegreen SL, et al. A multilevel ecological approach to promoting walking in rural communities. Prev Med. 2005;41(5-6):837-842. doi:10.1016/j.ypmed.2005.09.004

8. Brown WJ, Mummery K, Eakin E, Schofield G. 10,000 Steps Rockhampton: Evaluation of a Whole Community Approach to Improving Population Levels of Physical Activity. J Phys Act Health. 2006;3(1):1-14. doi:10.1123/jpah.3.1.1

9. Richardson AS, Ghosh-Dastidar M, Beckman R, et al. Can the introduction of a full-service supermarket in a food desert improve residents’ economic status and health? Ann Epidemiol. 2017;27(12):771-776. doi:10.1016/j.annepidem.2017.10.011

10. Joachim-Célestin M, Rockwood NJ, Clarke C, Montgomery SB. Evaluating the Full Plate Living lifestyle intervention in low-income monolingual Latinas with and without food insecurity. Womens Health. 2022;18:174550572210913. doi:10.1177/17455057221091350

11. Jago R, Mcmurray RG, Drews KL, et al. HEALTHY Intervention: Fitness, Physical Activity, and Metabolic Syndrome Results. Med Sci Sports Exerc. 2011;43(8):1513-1522. doi:10.1249/MSS.0b013e31820c9797

12. Wrigley N, Warm D, Margetts B. Deprivation, Diet, and Food-Retail Access: Findings from the Leeds ‘Food Deserts’ Study. Environ Plan Econ Space. 2003;35(1):151-188. doi:10.1068/a35150

13. Cummins S. Large scale food retailing as an intervention for diet and health: quasi-experimental evaluation of a natural experiment. J Epidemiol Community Health. 2005;59(12):1035-1040. doi:10.1136/jech.2004.029843

14. Cochrane T, Davey RC. Increasing uptake of physical activity: a social ecological approach. J R Soc Promot Health. 2008;128(1):31-40. doi:10.1177/1466424007085223

15. Ashfield-Watt P, Welch A, Godward S, Bingham S. Effect of a pilot community intervention on fruit and vegetable intakes: use of FACET (Five-a-day Community Evaluation Tool). Public Health Nutr. 2007;10(7):671-680. doi:10.1017/S1368980007382517

16. McCurley JL, Fortmann AL, Gutierrez AP, et al. Pilot Test of a Culturally Appropriate Diabetes Prevention Intervention for At-Risk Latina Women. Diabetes Educ. 2017;43(6):631-640. doi:10.1177/0145721717738020

17. Van Stappen V, Cardon G, De Craemer M, et al. The effect of a cluster-randomized controlled trial on lifestyle behaviors among families at risk for developing type 2 diabetes across Europe: the Feel4Diabetes-study. Int J Behav Nutr Phys Act. 2021;18(1):86. doi:10.1186/s12966-021-01153-4

18. Torrence C, Griffin SF, Rolke L, Kenison K, Marvin A. Faithful Families Cooking and Eating Smart and Moving for Health: Evaluation of a Community Driven Intervention. Int J Environ Res Public Health. 2018;15(9):1991. doi:10.3390/ijerph15091991

19. Aubrey-Bassler K, Fernandes C, Penney C, et al. The effectiveness of a proven chronic disease prevention and screening intervention in diverse and remote primary care settings: an implementation study on the BETTER 2 Program. BJGP Open. 2019;3(3):bjgpopen19X101656. doi:10.3399/bjgpopen19X101656

20. Simon C, Schweitzer B, Oujaa M, et al. Successful overweight prevention in adolescents by increasing physical activity: a 4-year randomized controlled intervention. Int J Obes.

21. Novotny R, Davis J, Butel J, et al. Effect of the Children’s Healthy Living Program on Young Child Overweight, Obesity, and Acanthosis Nigricans in the US-Affiliated Pacific Region: A Randomized Clinical Trial. JAMA Netw Open. 2018;1(6):e183896. doi:10.1001/jamanetworkopen.2018.3896

22. Ivester P, Sergeant S, Danhauer S, et al. Effect of a Multifaceted, Church-Based Wellness Program on Metabolic Syndrome in 41 Overweight or Obese Congregants. Cent Dis Control Prev. 2010;7(4):1-8.

23. Frediani JK, Li J, Bienvenida A, Higgins MK, Lobelo F. Metabolic Changes After a 24-Week Soccer-Based Adaptation of the Diabetes Prevention Program in Hispanic Males: A One-Arm Pilot Clinical Trial. Front Sports Act Living. 2021;3:757815. doi:10.3389/fspor.2021.757815

24. Chesla CA, Chun KM, Kwong Y, et al. Cultural Adaptation of the Group Lifestyle Balance Program for Chinese Americans. Diabetes Educ. 2016;42(6):686-696. doi:10.1177/0145721716666679

25. Andersen E, Burton NW, Anderssen SA. Physical activity levels six months after a randomised controlled physical activity intervention for Pakistani immigrant men living in Norway. Int J Behav Nutr Phys Act. 2012;9(1):47. doi:10.1186/1479-5868-9-47
